# The Proteome and Phosphoproteome Uncovers Candidate Proteins Associated With Vacuolar Phosphate Signal Multipled by Vacuolar Phosphate Transporter 1 (VPT1) in Arabidopsis

**DOI:** 10.1016/j.mcpro.2023.100549

**Published:** 2023-04-18

**Authors:** Yanjun Zhang, Xuexue Chen, Jinjing Feng, Yuanyue Shen, Yun Huang

**Affiliations:** Beijing Key Laboratory for Agricultural Application and New Technique, College of Plant Science and Technology, Beijing University of Agriculture, Beijing, China

**Keywords:** vacuolar phosphate transporter 1, proteome, phosphoproteome, Arabidopsis, phosphate signal

## Abstract

Plant vacuoles serve as the primary intracellular compartments for inorganic phosphate (Pi) storage. Passage of Pi across vacuolar membranes plays a critical role in buffering the cytoplasmic Pi level against fluctuations of external Pi and metabolic activities. To gain new insights into the proteins and processes, vacuolar Pi level regulated by vacuolar phosphate transporter 1 (VPT1) in Arabidopsis, we carried out tandem mass tag labeling proteome and phosphoproteome profiling of Arabidopsis WT and *vpt1* loss-of-function mutant plants. The *vpt1* mutant had a marked reduced vacuolar Pi level and a slight increased cytosol Pi level. The mutant was stunted as reflected in the reduction of the fresh weight compared with WT plants and bolting earlier under normal growth conditions in soil. Over 5566 proteins and 7965 phosphopeptides were quantified. About 146 and 83 proteins were significantly changed at protein abundance or site-specific phosphorylation levels, but only six proteins were shared between them. Functional enrichment analysis revealed that the changes of Pi states in *vpt1* are associated with photosynthesis, translation, RNA splicing, and defense response, consistent with similar studies in Arabidopsis. Except for PAP26, EIN2, and KIN10, which were reported to be associated with phosphate starvation signal, we also found that many differential proteins involved in abscisic acid signaling, such as CARK1, SnRK1, and AREB3, were significantly changed in *vpt1*. Our study illuminates several new aspects of the phosphate response and identifies important targets for further investigation and potential crop improvement.

## Highlights

• The proteome and phosphoproteome revealed that Pi signal in Arabidopsis vacuole is associated with photosynthesis, translation, RNA splicing, and phytohormone.
• Many differential proteins involved in abscisic acid were found.
• These finds illuminate new aspects of systemic vacuole Pi starvation signal network.

## Graphical Abstract

**Figure:**
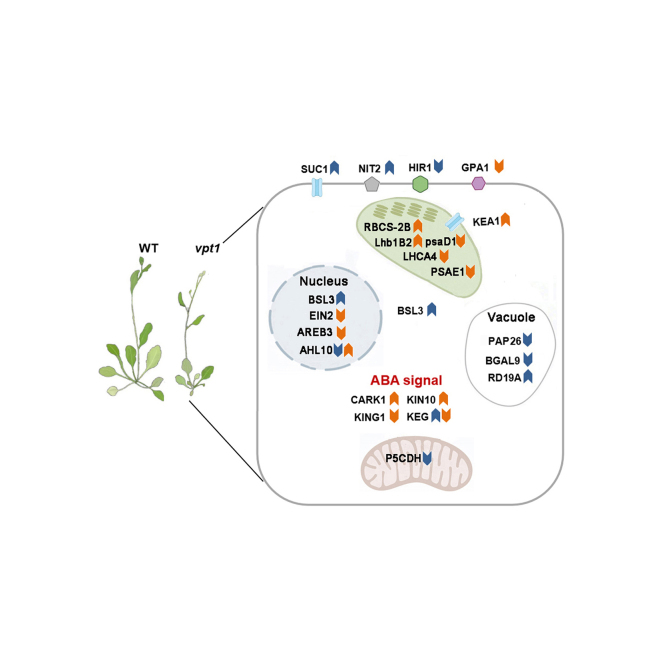
Summary of observed changes in protein abundance and protein phosphorylation in *vpt1* mutant.

## In Brief

The article not only illuminates several new aspects of systemic vacuole Pi starvation signal network but also identifies important targets for further investigation and potential crop improvement. This study could provide a cue that how the system phosphate signal affects photosynthesis, carbohydrate metabolic process, and plant hormone.

Inorganic phosphate (Pi) is an essential structural constituent of important biomolecules, such as nucleic acids, phospholipids, and sugar phosphates and also plays central roles in photosynthesis and respiration as well as in signal transduction and metabolic regulation *via* its covalent attachment to phosphoproteins. Pi sequestration into the vacuole plays a key role in temporary storage of cellular Pi and controls the fine-tuning of systemic Pi allocation, which is particularly important for reproductive development. The previous studies demonstrate that vacuolar phosphate transporter 1 (VPT1 or PHT5;1) contributes to Pi sequestration in the vacuole (1, 2). VPT1 is localized at the tonoplast, and loss of its function results in low vacuolar Pi content and impairs plant adaptation to changing Pi status in the environment (1).

The phosphate transporter VPT1/PHT5;1 plays an important role in plant growth and development (1, 2). The loss of function of VPT1 in plants displayed shorter primary roots (3), stunted vegetative growth as reflected in the reduction of the fresh weight (1), and bolting earlier under normal growth conditions in soil (3). Meanwhile, the *vpt1* mutant has a reduced vacuolar Pi level, but an increased cytosol Pi level (2), leading to Pi toxicity under high Pi condition. VPT1 is also an SPX (Syg1 [Suppressor of Yeast gpa1], Pho81 [yeast Phosphatase 81], and Xpr1 [human Xenotropic and Polytropic Retrovirus receptor 1]) domain–containing protein, which has been suggested to bind inositol hexaphosphate in a signaling pathway that senses cytosolic Pi levels and can regulate Pi redistribution (4).

Plants have shown a uniform response to phosphate deficiency by increasing starch synthesis relative to sucrose, but the accompanying limitation on photosynthetic capacity varies considerably among the species. Phosphate deficiency results in a significant decrease in the leaf Pi, diminished rate of photosynthesis, and a decrease in the sucrose/starch ratio in the leaves. Systemic or long-distance responses depend on the internal Pi concentration and participate in the overall enhancement of Pi uptake, reallocation, and recycling to ensure the metabolic balance of P at the whole-plant level (5). Changes in the cytoplasmic Pi content are relatively small in comparison with the large variations in vacuolar Pi on the phosphate-deficient medium (6).

There have been some reports on hormones related to phosphate stress response. Strigolactones, cytokinin, and auxin signaling act as phosphate stress response hormone that regulate root and root hair elongation to enlarge the root absorbing surface and to increase Pi concentrations in roots (7, 8, 9). Low phosphate significantly activated plant ABA biosynthesis, metabolism, and stress responses and promoted Pi uptake in an ABA-INSENSITIVE 5 (ABI5)–dependent manner in *Arabidopsis thaliana* (10). However, the clear phosphate-deficient signal transduction of vacuolar Pi, especially the hormones involved, remains unknown.

The aim of the present study is to exploit high-resolution liquid chromatography–tandem mass spectrometry (LC–MS/MS) to obtain a comprehensive overview of the impact of vacuolar Pi level on the proteome and phosphoproteome of the model plant *A. thaliana*. This study attempts to provide a cue that how the vacuole concentration of Pi affects phytohormone and photosynthesis. This approach allowed us to elucidate cellular responses that are reliant on vacuolar Pi state. Our study also helps to better define the molecular mechanisms by which vacuolar Pi-deficient signal transmits to other organelles and plant cells, which holds promise for potential benefits in agricultural biotechnology.

## Experimental Procedures

### Plant Materials

Arabidopsis Col-0 WT and *vpt1* plants were grown in soil at the Beijing University of Agriculture in an environmentally controlled chamber under long day conditions (16 h light/8 h dark cycle) and controlled conditions as described (3); including air temperature of 22 ^°^C, air humidity of 70%, and an incident light intensity of 220 mmol/m^2^/s at the plant level. At 30th day after sowing, we harvested aerial part from three independent biological replicates for proteome and phosphoproteome analyses.

### Experimental Design and Statistical Rationale

**Fig. 1:**
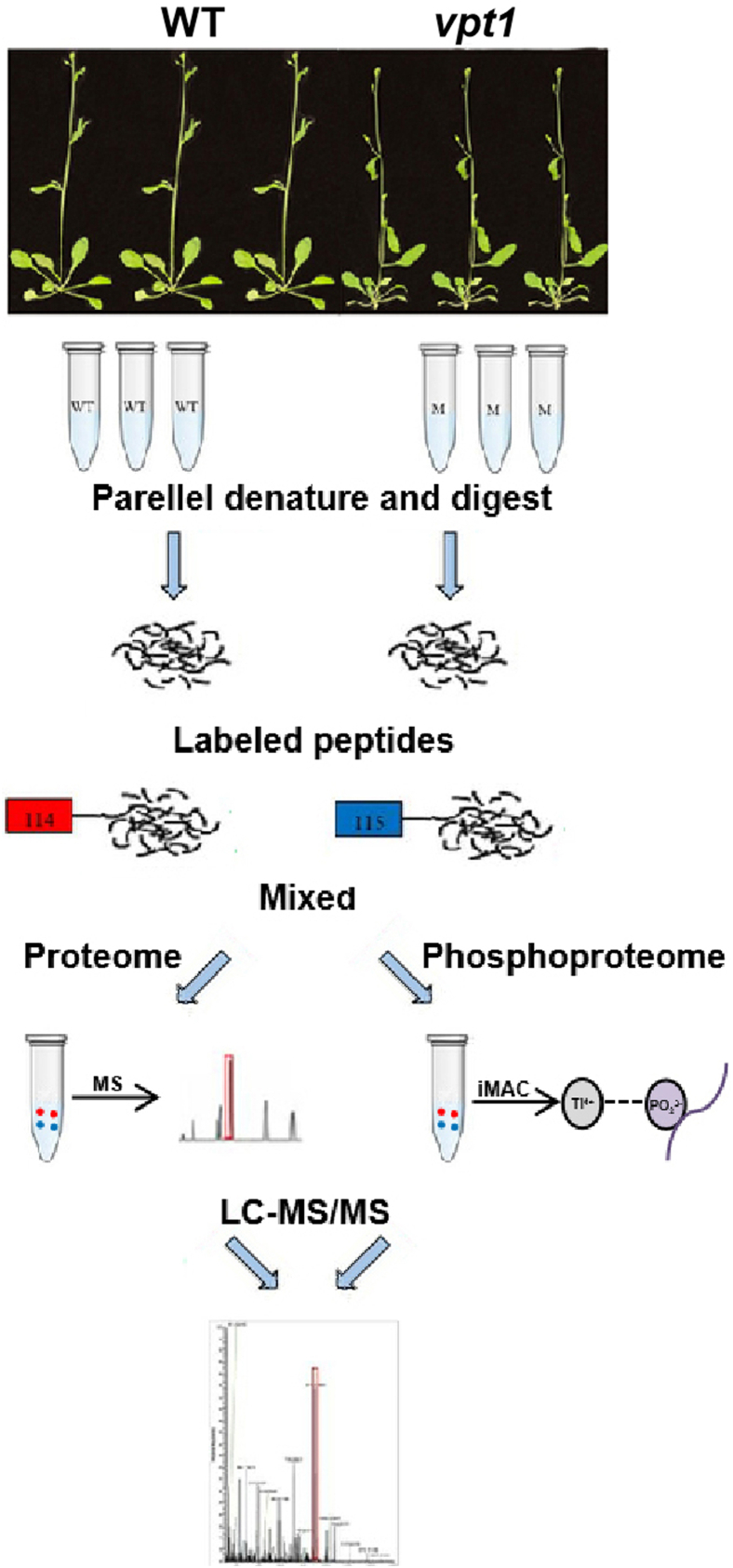
**Schematic representation of the experimental workflow.** The aerial part of Arabidopsis WT and *vpt1* plants grown in soil at 30th day after sowing (DAS) was harvested with three independent biological replicates. Protein samples were labeled with individual tandem mass tag (TMT) reagents for proteome and phosphoproteome analyses.

The proteome and phosphoproteome of the samples were studied by tandem mass tag (TMT) labeling, HPLC fractionation, phosphopeptide enrichment, and a series of techniques. The workflow is shown in Figure 1. Two groups of samples (WT and *vpt1*) were detected, with three biological replicates in each group, a total of six samples. We used principal component analysis, relative standard deviation, and Pearson's correlation coefficient to evaluate the repeatability of protein quantification/modification quantification. The analysis of proteome and phosphoproteome data is based on a 1.2-fold threshold for differential expression changes, with *t* test *p* value < 0.05 as the significance threshold. Phosphoproteome data were filtered according to the standard (localization probability >0.75) and normalized with the protein quantification group to remove the effect of protein expression on the modification signal. Quantitative proteins/quantitative sites indicate that at least one comparative group has quantitative information. Protein annotations were performed in terms of Gene Ontology (GO), protein domain, Kyoto Encylopedia of Genes and Genomes pathway, and subcellular structure localization. The differential protein interaction network was displayed by R package “networkD3” (STRING, version 10.5, confidence score >0.7).

### Proteome Analysis

#### Protein Extraction

The sample was grinded by liquid nitrogen into cell powder and then transferred to a 5 ml centrifuge tube. After that, four volumes of lysis buffer (8 M urea, 1% Triton-100, 10 mM dithiothreitol, and 1% Protease Inhibitor Cocktail) were added to the cell powder, followed by sonication three times on ice using a high-intensity ultrasonic processor (Scientz). The remaining debris was removed by centrifugation at 20,000*g* for 10 min at 4 °C. Finally, the protein was precipitated with cold 20% trichloroacetic acid for 2 h at −20 °C. After centrifugation at 12,000*g* for 10 min at 4 °C, the supernatant was discarded. The remaining precipitate was washed with cold acetone for three times. The protein was redissolved in 8 M urea, and the protein concentration was determined with bicinchoninic acid kit according to the manufacturer’s instructions.

#### Trypsin Digestion

For digestion, the protein solution was reduced with 5 mM dithiothreitol for 30 min at 56 °C and alkylated with 11 mM iodoacetamide for 15 min at room temperature in darkness. The protein sample was then diluted by adding 100 mM triethylamine bicarbonate until the urea concentration was less than 2 M. Finally, trypsin was added at 1:50 trypsin-to-protein mass ratio for the first digestion overnight and 1:100 trypsin-to-protein mass ratio for a second 4 h digestion.

#### TMT Labeling

After trypsin digestion, peptide was desalted by Strata X C18 SPE column (Phenomenex) and vacuum dried. Peptide was reconstituted in 0.5 M triethylamine bicarbonate and processed according to the manufacturer’s protocol for TMT kit. Briefly, one unit of TMT reagent was thawed and reconstituted in acetonitrile. The peptide mixtures were then incubated for 2 h at room temperature and pooled, desalted, and dried by vacuum centrifugation.

#### HPLC Fractionation

The tryptic peptides were fractionated into fractions by high pH reverse-phase HPLC using Agilent 300Extend C18 column (5 μm particles, 4.6 mm ID, 250 mm length). Briefly, peptides were first separated with a gradient of 8% to 32% acetonitrile (pH 9.0) over 60 min into 60 fractions. Then, the peptides were combined into 18 fractions and dried by vacuum centrifuging.

#### LC–MS Analysis

The tryptic peptides were dissolved in 0.1% formic acid (solvent A) and directly loaded onto a home-made reversed-phase analytical column (15 cm length, 75 μm i.d.). The gradient was comprised of an increase from 6% to 23% solvent B (0.1% formic acid in 98% acetonitrile) over 26 min, 23% to 35% in 8 min, and climbing to 80% in 3 min then holding at 80% for the last 3 min, all at a constant flow rate of 400 nl/min on an EASY-nLC 1000 ultraperformance liquid chromatography system.

The peptides were subjected to nanoelectrospray ionization source followed by MS/MS in Q Exactive Plus (Thermo) coupled online to the ultraperformance liquid chromatography. The electrospray voltage applied was 2.0 kV. The *m/z* scan range was 350 to 1800 for full scan, and intact peptides were detected in the Orbitrap at a resolution of 70,000. Peptides were then selected for MS/MS using normalized collision energy setting as 28, and the fragments were detected in the Orbitrap at a resolution of 17,500. A data-dependent procedure that alternated between one MS scan followed by 20 MS/MS scans with 15.0 s dynamic exclusion. Automatic gain control was set at 5E4. Fixed first mass was set as 100 *m/z*.

The resulting MS/MS data were processed using MaxQuant search engine (version 1.5.2.8) (http://www.maxquant.org/). Tandem mass spectra were searched against UniProt database concatenated with reverse decoy database. Trypsin/P was specified as cleavage enzyme allowing up to two missing cleavages. The mass tolerance for precursor ions was set as 20 ppm in first search and 5 ppm in main search, and the mass tolerance for fragment ions was set as 0.02 Da. Carbamidomethyl on Cys was specified as fixed modification, and oxidation on Met was specified as variable modifications. False discovery rate (FDR) was adjusted to <1%, and minimum score for peptides was set to >40. The isolation window at MS1 filtration is 2.0 *m/z* of proteome, and the isolation window at MS1 filtration is 1.4 *m/z* of phosphoproteome. MS-identified information of proteome is listed in Supplemental Table S1.

#### Database Search

The output MS/MS data were processed using MaxQuant search engine (version 1.5.2.8). The download/release date of the UniProt *A. thaliana* database is 201,804. Search parameters were set: the database was UniProt *A. thaliana* (39,369 sequences), inverse libraries were added to calculate the false positive rate because of random matches (FDR), and a common contamination library was added to the database to remove the effect of contaminating proteins from the identification results. The digestion method was set to Trypsin/P. The number of missed cut sites was set to two. The minimum length of the peptide was set to seven amino acid residues. The maximum number of peptide modifications was set to five. The mass error tolerance of primary parent ion for first search and main search was set to 20 ppm and 5 ppm, respectively. The mass error tolerance of the primary parent ion was set to 20 ppm and 5 ppm for first search and main search, and the mass error tolerance of the secondary fragment ion was set to 0.02 Da. The fixed modifications and variable modifications were oxidation of methionine, acetylation of N-terminal protein, and deamidation of asparagine phosphorylation of serine, threonine, and tyrosine. The quantification method was set to TMT-6plex. The FDR of protein identification and peptide-spectrum match identification was set to 1%.

### Phosphoproteome Analysis

#### Biomaterial-Based Post-Translational Modification Enrichment (for Phosphorylation)

Peptide mixtures were first incubated with immobilized metal affinity chromatography (IMAC) microsphere suspension with vibration in loading buffer (50% acetonitrile/6% trifluoroacetic acid). The IMAC microspheres with enriched phosphopeptides were collected by centrifugation, and the supernatant was removed. To remove nonspecifically adsorbed peptides, the IMAC microspheres were washed with 50% acetonitrile/6% trifluoroacetic acid and 30% acetonitrile/0.1% trifluoroacetic acid, sequentially. To elute the enriched phosphopeptides from the IMAC microspheres, elution buffer containing 10% NH_4_OH was added and the enriched phosphopeptides were eluted with vibration. The supernatant containing phosphopeptides was collected and lyophilized for LC–MS/MS analysis. MS-identified information of phosphoproteome is listed in Supplemental Table S2.

#### Bioinformatics Analysis

Localization predication soft: Wolfpsort v.0.2 (http://www.genscript.com/psort/wolf_psort.html). GO annotation: InterProScan (http://www.ebi.ac.uk/interpro/). Motif analysis: MoMo (http://meme-suite.org/tools/momo).

#### RNA Extraction and RT–Quantitative PCR

Total RNA was extracted from *vpt1* mutant and WT seedlings using Megan Biotech's HiPure HP Plant RNA Mini Kit. Total RNA (1.0 μg) was taken for reverse transcription, and detailed operation steps were performed according to the instructions. Quantitative real-time PCR (RT–qPCR) was performed using the TransStart Top Green qPCR SuperMix kit (TransGen Biotech) and the CFX96 Touch real-time PCR instrument (Bio-Rad). The internal control was *ACTIN2* (At3g18780). The gene-specific qPCR primers are shown in Supplemental Table S3.

## Results and Discussion

**Supplemental Figure S1:**
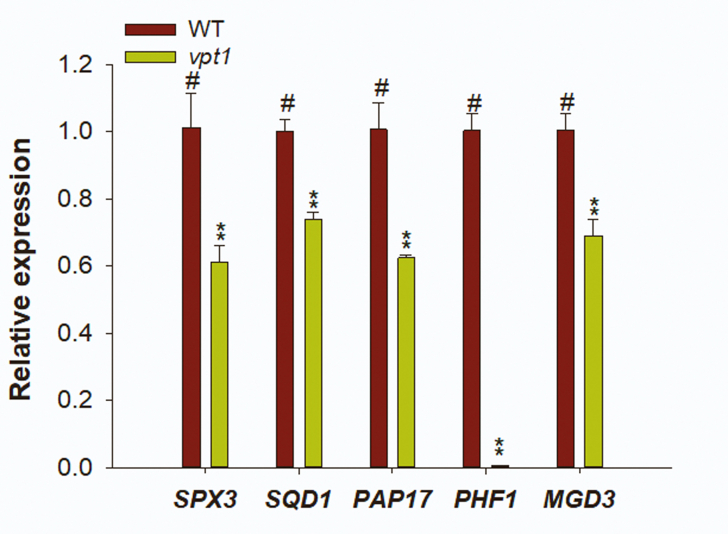
**Relative expression of phosphate starvation induced (*PSI*) genes.** Relative expression of *SPX3*, *SQD1*, *PAP17*, *PHF1*, and *MGD3* in *vpt1* mutant and WT seedlings was detected by RT-qPCR. Three biological replicates were tested for all samples. *ACTIN2* (At3g18780) was used as an internal control. The relative expression levels were calculated using the 2^−△△Ct^ method. The gene-specific qPCR primers are shown in Supplemental Table S3. The expression of typical *PSI* genes in vpt1 mutant was lower than that of WT.

**Supplemental Figure S2:**
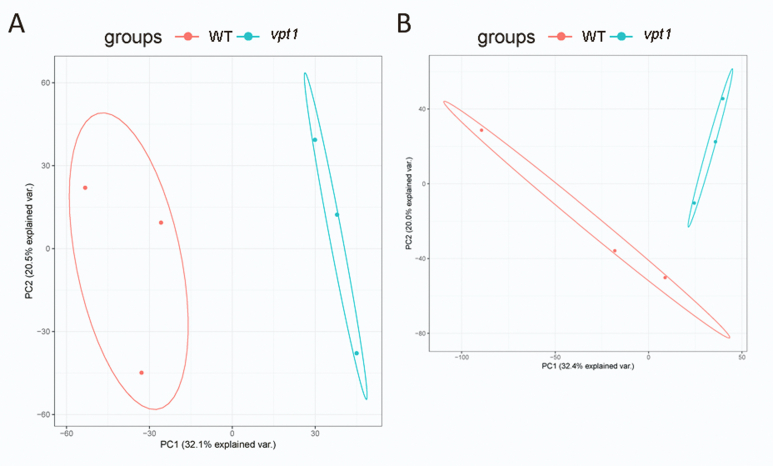
**PCA analysis of the proteomics and phosphoproteomics data.** The control samples (WT) and the *vpt1* mutants are well separated on the PCA plots in both the global proteome and the phosphoproteome analyses.

In this study, we performed quantitative proteomics and phosphoproteome of Arabidopsis seedlings WT as well as *vpt1* mutant plants, whose content of Pi in vacuoles was lower than that of WT, and the cytoplasmic Pi content was slightly higher than that of WT. Therefore, many phosphate starvation–induced (*PSI*) genes previously reported were reduced by cytoplasmic phosphate signals (1, 11). The results of RT–qPCR showed that the expression of typical *PSI* genes in *vpt1* mutant was lower than that of WT (Supplemental Fig. S1), which was consistent with previous reports (11). In order to reveal the changes in protein abundance and phosphorylation state caused by the mutation of *VPT1*, our simple workflow is shown in Figure 1. Each plant material contains three biological replicates. The quantitative values of (phospho)peptide abundance were derived by peak intensity. Principal component analysis of proteomics and phosphoproteomics data showed a clear separation between the WT and the *vpt1* mutant plants (Supplemental Fig. S2).

### Proteome and Phosphoproteome Analyses of Arabidopsis *vpt1* Mutant

**Fig. 2:**
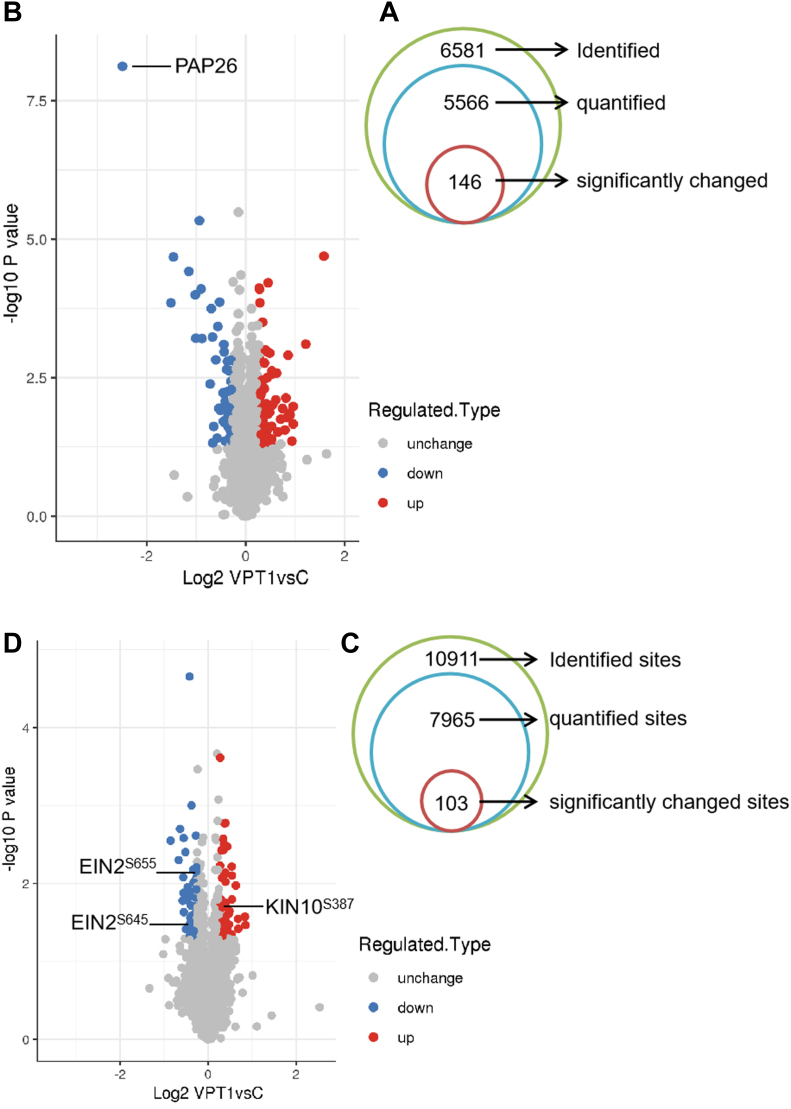
**Identification of significantly changed proteins in proteome and phosphoproteome.***A*, number of total quantified and significantly changed proteins in proteome. *Green cycle*: 6581 proteins have been identified from the nonmodified proteome analysis; *Blue cycle*: 5566 proteins had tandem mass tag (TMT) intensity values greater than zero; *Red cycle*: 146 proteins have been identified that were altered in abundance in the *vpt1* mutant plants. The significantly changed proteins were determined using the cutoff of fold change >1.2 or <0.83 and adjusted *p* value <0.05. *B*, Volcano plots showing upregulated and downregulated proteins in the *vpt1* mutant plants. *Blue points*: abundance decreased; *Red points*: abundance increased. *X*-axis is the Log2 of fold change (*vpt1*/WT), and *Y*-axis is the negative Log10 of the *p* value for independent *t* test adjusted. *C*, number of total quantified and significantly changed proteins in phosphoproteome. *Green cycle*: 10,911 phosphorylation sites have been identified; *Blue cycle*: 7965 phosphorylation sites have been quantified from the phosphopeptide-enriched material; *Red cycle*: 103 phosphorylation sites had significantly changed as they had an adjusted *p* value < 0.05 and fold change (FC) >1.2 or <0.83. *D*, Volcano plots showing upregulated and downregulated phosphopeptides in the *vpt1* mutant plants. *Blue points*: phosphorylation level decreased; *Red points*: phosphorylation level increased. *X*-axis is the Log2 of FC (*vpt1*/WT), and *Y*-axis is the negative Log10 of the *p* value for independent *t* test adjusted. The changed phosphorylation sites of EIN2 and KIN10 were labeled.

We identified 6581 proteins from the nonmodified proteome analysis. For further quantitative analyses, we focused on the 5566 protein groups that had TMT intensity values greater than zero (Supplemental Table S4). Next, we identified proteins that were altered in abundance in the *vpt1* mutant plants (Fig. 2*A*). For these statistical analyses, we used the software package Perseus (Maxquant Company) to calculate two-sample *t* tests and perform permutation-based FDR correction. We designated that 146 of these proteins were significantly changed in abundance as they had an adjusted *p* value < 0.05 and fold change >1.2 or <0.833 (Fig. 2*A* and Supplemental Table S5). This revealed 52 proteins that decreased and 94 proteins that increased in abundance in the *vpt1* mutant relative to WT plants (Fig. 2*B*).

In addition, we quantified 7965 phosphorylation sites arising from 3566 phosphoproteins (localization probability ≥0.75) from the phosphopeptide-enriched material (Fig. 2*C* and Supplemental Table S6). Examination of the identified phosphoproteins revealed 1703 (21%) represented newly identified phosphorylation sites when compared with the compendium of 79,334 known phosphorylation sites (PhosPhat 4.0; (12)). After normalization with protein quantitation group to remove the effect of protein expression on the modified signal, 3288 phosphorylation sites on 1312 proteins were obtained. We designated significantly changed 103 phosphorylation sites on 83 proteins as they had an adjusted *p* value < 0.05 and fold change >1.2 or <0.833 (Supplemental Table S7). Among them, 50 phosphorylation sites decreased and 53 sites increased in phosphorylation signal in the *vpt1* mutant (Fig. 2*D*). Taken together, these results demonstrated extensive alteration of proteome and phosphoproteome composition in *vpt1* plants whose content of phosphate in vacuoles was lower than that of WT.

Among the 146 differential abundance proteins (DAPs), a well-characterized protein is the specific vacuolar and secreted purple acid phosphatases (PAPs) (13). PAPs have mostly been studied for their potential involvement in phosphorus acquisition and redistribution because of their ability to catalyze the hydrolysis of activated phosphate esters and anhydrides under acidic conditions. We detected significant decreases in the abundance of local Pi-starvation–inducible PAP26 (At5g34850; Fig. 2*B*), suggesting possible specificity in the regulation compared with other PAPs.

In our study, the abundance of ETHYLENE-INSENSITIVE2 (EIN2; At5g03280) was increased in the *vpt1* mutant relative to WT (Supplemental Table S4), and the phosphorylation level of Ser645 and Ser655 in EIN2 was significantly decreased in *vpt1* mutant (Supplemental Table S7). EIN2, a positive regulator of ethylene signaling, is stabilized at the protein level and activated by dephosphorylation in the presence of ethylene, which protects it from proteasomal degradation (14, 15). The site Ser645 of EIN2 is phosphorylated by CONSTITUTIVE TRIPLE RESPONSE 1 (CTR1), which leads to EIN2 degradation and prevents EIN2-C from translocation into the nucleus (14, 16). Ser655 has not been reported. However, the adjacent site Ser657 is phosphorylated by TARGET OF RAPAMYCIN, thus forming glucose-TARGET OF RAPAMYCIN–EIN2 pathway that is decoupled from canonical ethylene-CTR1–EIN2 signaling (17). Our analysis result of EIN2 protein in *vpt1* mutant is consistent with previous study that ethylene signaling positively regulates phosphate starvation response in Arabidopsis (18).

The phosphorylation level of sucrose nonfermenting 1-related protein kinase (SnRK1) catalytic subunit alpha (KIN10; At3g01090) was increased in the *vpt1* mutant. Previous study has showed that the *kin10* mutant plants are deficient in starch mobilization at night during Pi starvation, and SnRK1 activity in transgenic plants overexpressing *KIN10* is increased by 100%, which indicates an important role of KIN10 in signaling during phosphate starvation (19).

The results suggested that ethylene signaling was enhanced because of the low phosphate content in the *vpt1* mutant. Except for aforementioned PAP26, EIN2, and KIN10, the majority of differential proteins in proteome and phosphoproteome have not previously been reported to be involved in phosphate signal network.

### Identification of DAPs and Phosphoproteins in Arabidopsis *vpt1* Mutant

**Fig. 3:**
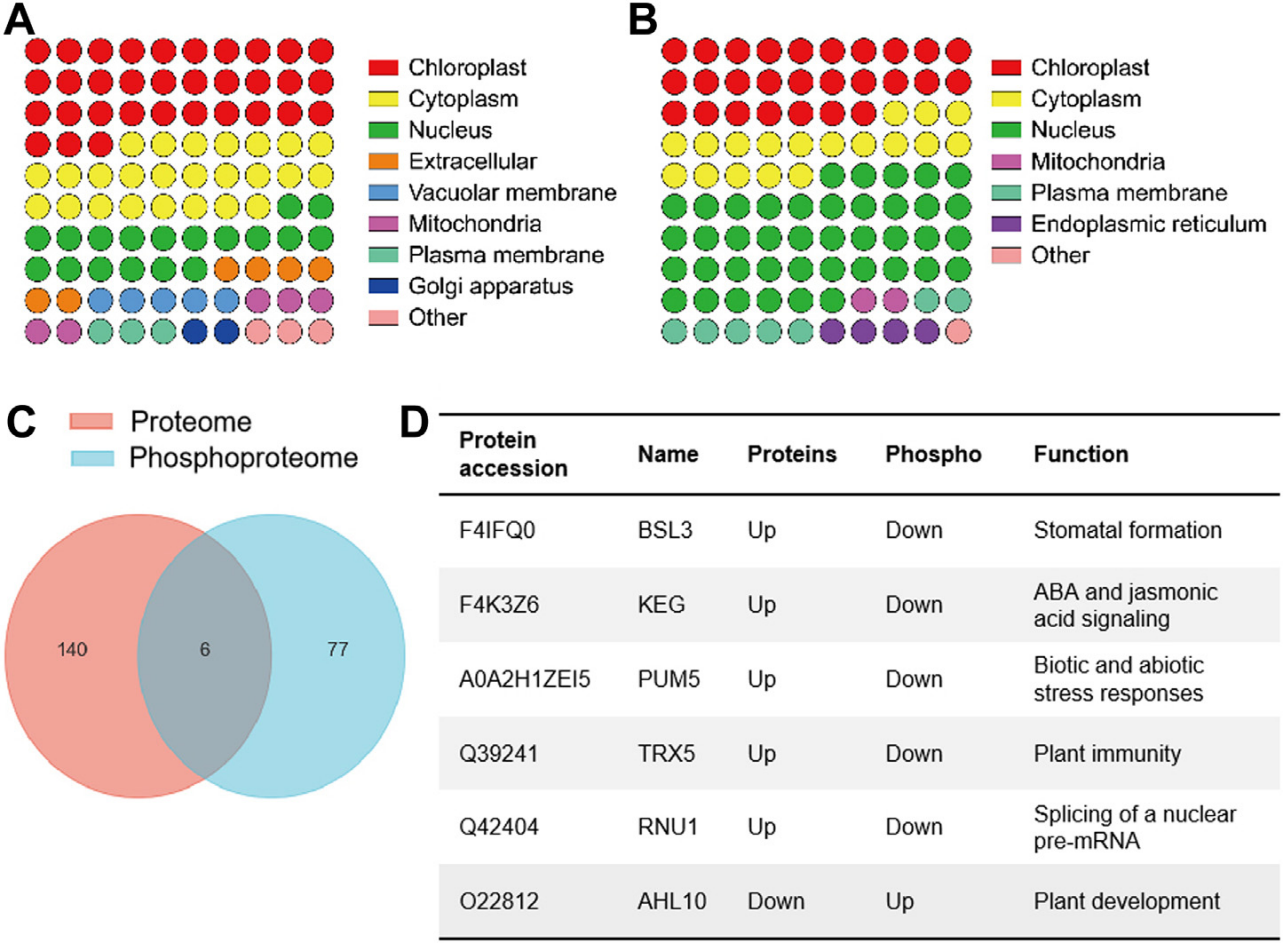
**Subcellular localization predication and overlap of significantly changed proteins in proteome and phosphoproteome.***A*, the subcellular compartmentalization of differential abundance proteins (DAPs). The *different colored circles* represent the ratio of different subcellular locational proteins to total DAPs. *B*, the subcellular compartmentalization of DAPs and phosphoproteins (DAPPs). The *different colored circles* represent the ratio of different subcellular locational proteins to total DAPPs. *C*, Venn diagram showing numbers of significantly changed proteins in proteome and phosphoproteome and overlapped proteins. *D*, the information of six overlapped proteins between proteome and phosphoproteome. The proteins included BSL3, KEG, PUM5, TRX5, RNU1, and AHL10.

We also determined the subcellular compartmentalization of DAPs and phosphoproteins (DAPPs) using the consensus localization predication soft Wolfpsort v.0.2 (20). DAPs and DAPPs exhibited a similar distribution of localizations, mainly in nucleus, chloroplast, and cytoplasm (Fig. 3, *A* and *B*). It can be seen that seven vacuole DAPs have been determined in the proteomic profile (4.7%; Fig. 3*A*). It is worth noting that only beta-galactosidase9 (BGAL9; At2g32810), a cell wall–bound enzyme known to degrade the wall polysaccharides related to photosynthesis, and PAP26 were significantly decreased, but responsive to dethydration19A (RD19A; At4g39090), CAMV movement protein interacting protein7 (PRA1D; At1g04260), vacuolar sorting receptor4 (VSR4; At2g14720), cyclase1 (CYCLASE1; At4g34180), and aspartyl protease (AED1; At5g10760) were increased in protein abundance. VSR4 was indicated to be a transmembrane protein that mediates vacuole storage protein transport *via* specific protein–protein interactions (21).

All the 83 proteins exhibiting a significant change in phosphorylation (Supplemental Table S7) were quantified in our proteome data (Supplemental Table S4). We then directly compared the significantly changed phosphoproteome and proteome to identify proteins exhibiting a change in both protein abundance and phosphorylation status. We found that a total of six phosphoproteins (4.1% of all 146 proteins significantly changing in protein abundance) fit these criteria (Fig. 3*C*). These included BRI1 SUPPRESSOR 1 (BSU1)-LIKE 3 (BSL3; At2g27210), KEEP ON GOING (KEG; At5g13530), Arabidopsis Pumilio RNA-binding protein 5 (PUM5/APUM5; At3g20250), THIOREDOXIN H-TYPE 5 (TRX5; At1g45145), U1 SMALL NUCLEAR RIBONUCLEOPROTEIN-70K (RNU1; At3g50670), and AT-HOOK MOTIF NUCLEAR LOCALIZED PROTEIN 10 (AHL10; At2g33620). These six overlapped proteins of phosphoproteome and proteome exhibited opposing patterns of phosphorylation and abundance changes, suggesting that phosphorylation may impact their turnover (Fig. 3*D*).

BSL3 is one of a four-member gene family BSU1-Like in Arabidopsis. It is reported either negative regulators or positive regulators of stomatal production depending on whether it is combined with BSL1 (At4g03080) or BSU1 (At1g03445), respectively. When combined with BSL1, BSL3 confers a negative regulation, whereas when combined with BSU1, BSL3 confers a positive regulation (22). KEG encodes a RING-type E3 ligase that modulates abscisic acid (ABA) signaling by regulating the protein level of ABI5 (At2g36270) (23) and jasmonic acid signaling by mediating JASMONATE ZIM-DOMAIN12 stability (24). Arabidopsis Pumilio RNA-binding protein 5 functions as a negative regulator under abiotic stress by directly binding to the 3′ UTR of target genes (25). THIOREDOXIN H-TYPE 5 is an oxidoreductase that reversed SNO modifications by acting as an SNO reductase in plant immunity (26). U1 SMALL NUCLEAR RIBONUCLEOPROTEIN-70K participates in alternative splicing of pre-mRNAs (27) and may be involved in response to osmotic stress (28). AHL10, a DNA-binding protein, was dephosphorylated by highly ABA-induced1 (At5g59220) to coordinate growth with stress and defense responses (29). Further investigation of these protein post-translational regulation in phosphate signal pathway is needed.

### Proteins of Photosynthesis and Hormone Signal Responses are Significantly Changed in *vpt1* Mutant

Disruption of PHT5;1/VPT1 reduces total Pi content in the plant, leading to a marked reduction in vacuoles and slight increase in cytoplasm (11). The changes in proteome and phosphoproteome in the *vpt1* mutant may be the consequence of the alteration in vacuolar Pi levels or the increased cytosolic Pi level.

**Fig. 4:**
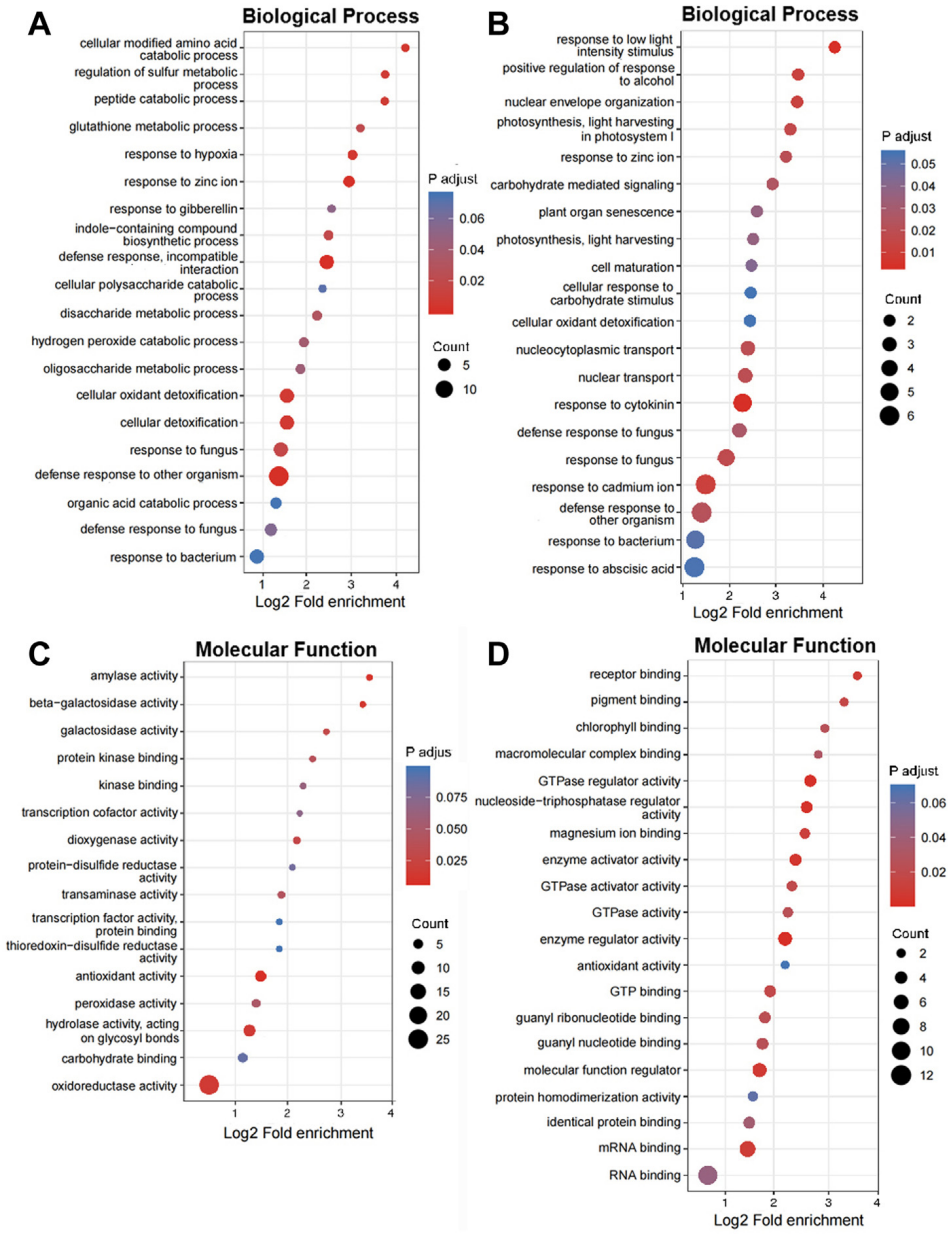
**Functional enrichment analyses of DAPs and DAPPs.***A* and *B*, Gene Ontology (GO) terms of biological process of DAPs (*A*) and DAPPs (*B*). *C* and *D*, GO terms of molecular function of DAPs (*C*) and DAPPs (*D*). DAP, differential abundance protein; DAPP, DAP and phosphoprotein.

To gain insight into the processes that are impacted by the mutation of *vpt1*, we performed GO enrichment analyses. According to the functions or putative functions, the DAPs and DAPPs were both distributed into 20 biological processes (Fig. 4, *A* and *B* and Supplemental Tables S9 and S10). Consistent with a deficiency in vacuole Pi in the mutants, GO terms, such as photosynthesis and carbohydrate-mediated signaling, known to be impacted by phosphorus, are overrepresented among proteins that were changed in abundance and phosphorylation levels. The different abundance proteins involved in carbohydrate metabolic process including bifunctional UDP-glucose 4-epimerase and UDP-xylose 4-epimerase 1 (UGE1; At1g12780), glutamine-fructose-6-phosphate transaminase 2 (GFAT; At3g24090), beta-1,3-glucanase 2 (BGL2; At3g57260), beta-galactosidase 4 (BGAL4; At5g56870), and ATP-dependent 6-phosphofructokinase 4 (PFK4; At5g61580), starch catabolic process alpha-amylase 1 (AMY1; At4g25000), and sucrose metabolic process including sucrose transport protein (SUC1; At1g71880) and sucrose synthase (SUS6; At1g73370) were all increased except BGAL9 and beta-amylase3 (BAM3; At4g17090) (30). The phosphorylation levels of proteins from carbohydrate metabolic process glucose-6-phosphate 1-dehydrogenase (G6PD5; At3g27300) and photosynthesis including chlorophyll a–b binding protein (Lhb1B2; At2g34420), photosystem I reaction center subunit II-1 (psaD1; At4g02770), chlorophyll a–b binding protein 4 (LHCA4; At3g47470), ribulose bisphosphate carboxylase small chain 2B (RBCS-2B; At5g38420), photosystem I reaction center subunit IV A (PSAE1; At4g28750) were significantly changed. These results suggest that the mutation of *VPT1*/*PHT5;1* trigger Pi response protein reprogramming.

Many proteins are connected to plant hormone ABA, gibberellin, jasmonic acid, cytokinin, and auxin signaling. Namely, GO terms related to phytohormones were enriched (Fig. 4, *A* and *B* and Supplemental Tables S9 and S10). Consistent with the dwarf and bolting earlier phenotype for *vpt1*, growth-promoting phytohormones were enriched. Associated with gibberellin signaling, gibberellin-regulated protein 7 (GASA7; At2g14900), which may function in hormonal-controlled biological processes, such as seed germination, flowering, and seed maturation (31), was enriched with higher levels in *vpt1*. Indicator of auxin biosynthesis, nitrilase 2 (NIT2; At3g44300), which catalyze indole-3-acetonitrile into auxin indole-3-acetic acid and positively regulate flowering (32), was increased in the *vpt1* mutant compared with WT.

Stress response–related phytohormones were also enriched. Methylesterase 9 (MES9; At4g37150) and allene oxide cyclase 3 (AOC3; At3g25780) involved in jasmonic acid metabolic process were significantly enriched in *vpt1*. The proteins with significant changes were related to ABA. Cytosolic ABA receptor kinase1 (CARK1; At3g17410), a receptor-like cytoplasmic kinase, that acts as a novel positive regulator of ABA signaling by phosphorylating ABA receptors PYL8/RCAR3 (At5g53160) and PYR1/RCAR11 (At4g17870) (33), was increased in phosphorylation level. Guanine nucleotide-binding protein alpha-1 subunit (GPA1; At2g26300) was decreased in phosphorylation level. Stomatal closure in *gpa1* plants was hyposensitive to ABA (34).

The abundance of SnRK1 regulatory subunit gamma 1 (KING1; At3g48530) was lower, whereas the phosphorylation level of SnRK1 catalytic subunit alpha KIN10 was increased in *vpt1*. The SnRK complex not only plays a central role in sugar and ABA signaling (35) but also is involved in the regulation of fatty acid synthesis by phosphorylation of acetyl-CoA carboxylase and in assimilation of nitrogen by phosphorylating nitrate reductase (36). The bZIP family transcription factor (TF) ABI5-like protein 2 (DPBF3/AREB3; At3g56850) was enriched with lower phosphorylation level, which binds to the embryo specification element and the ABA-responsive element of the *Dc3* gene promoter and could participate in ABA-regulated gene expression during seed development (37). DPBF3 binds to *AtSUC1* promoter and potentially regulates its expression in young seedlings, indicated the complex interactions between ABA and sugar signaling networks (38). These results further demonstrate that the mutation of VPT1/*PHT5;1* induces significant changes of ABA signaling. This information can benefit future studies in Pi signal transduction.

### Significantly Changed Protein Kinases and the Phosphorylated Motif Analysis

According to the GO enrichment analyses of molecular function, many proteins were changed in proteome and phosphoproteome (Fig. 4, *C* and *D* and Supplemental Tables S9 and S10). Seven protein kinases including three at the protein abundance levels (Table 1) and four at the phosphopeptide levels were found to be changed in *vpt1* plants (Table 2).

**Table 1:** Different abundance protein kinases in proteome

Protein accession	Protein description	Gene name	VPT1/WT ratio	VPT1/WT *p* value
O49339	PTI1-like tyrosine-protein kinase 2	PTI12	1.399	0.0011403
Q9FM19	Hypersensitive-induced response protein 1	HIR1	0.739	0.00108184
Q9SJQ0	Pyruvate kinase	At2g36580	1.213	0.00008

*vpt1*/WT ratio: Protein content ratio of *vpt1* mutant to WT.

*p* < 0.05 indicates significant difference.

**Table 2:** Different abundance protein kinases in phosphoproteome

Protein accession	Protein description	Gene name	VPT1/WT ratio	VPT1/WT *p* value	Amino acid
F4II29	Kinase family with ARM repeat domain–containing protein	CTEXP	1.38	0.024583	S605
Q38997	SNF1-related protein kinase catalytic subunit alpha KIN10	KIN10	1.213	0.0177351	S387
Q9LJD8	MAP3K epsilon protein kinase 1	M3KE1	0.773	0.037764	S434
Q9LUT0	Protein kinase superfamily protein	CARK1	1.297	0.034401	S79
Q9LUT0	Protein kinase superfamily protein	CARK1	1.266	0.028904	T358

Abbreviations: S, serine; T, threonine; *vpt1*/WT Ratio, Phosphoprotein content ratio of *vpt1* mutant to WT.

*p* < 0.05 indicates significant difference.

Amino acid column represents the corresponding phosphorylation sites.

In terms of protein abundance, PTI12 (PTI1-like tyrosine-protein kinase 2), a Ser/Thr kinase activated by convergent phosphatidic acid and oxidative stress signaling pathways (39), and pyruvate kinase (At2g36580) involved in seed oil biosynthesis (40) were increased. Moreover, hypersensitive-induced response protein1 (HIR1; At5g62740), which is a positive regulator of hypersensitive response–like cell death and may be involved in potassium ion channel regulation (41), was decreased. In addition, beside KIN10 and CARK1 in ABA signaling, the phosphorylation level of CYTOPLASMIC TRNA EXPORT PROTEIN (CTEXP; At2g40730) increased, and transcriptome analysis showed that CTEXP regulated pollen germination and tube growth (31). MAP3K EPSILON PROTEIN KINASE (M3KE1/MAPKKK7; At3g13530), a serine/threonine-protein kinase involved in the spatial and temporal control system organizing cortical activities in mitotic and postmitotic cells (42, 43), was decreased.

Motif analysis was performed on DAPPs using the MoMo algorithm. Four motifs including RXXS, SP, and SDXE motifs were significantly enriched in *vpt1* mutant. The result is consistent with the significant changed protein kinases. The RXXS motif is an extremely common motif targeted by SNF1-related kinase, calcium-dependent protein kinase (CDPK), calmodulin-dependent protein kinase, and CBL-interacting protein kinase (44, 45, 46, 47). Proline-directed motif SP is also an extremely common motif as a potential substrate for MAPK, CDPK, SnRK2, and RLK (48). SDXE motif is acidic S-type targeted by SnRK1, CDPK, and casein kinase II (45, 46). According to the significantly changed protein kinases and the motif analysis, it suggests that potential SnRKs and MAPK cascade pathways are responsible for Pi homeostasis in Arabidopsis.

### Protein–Protein Interaction

**Fig. 5:**
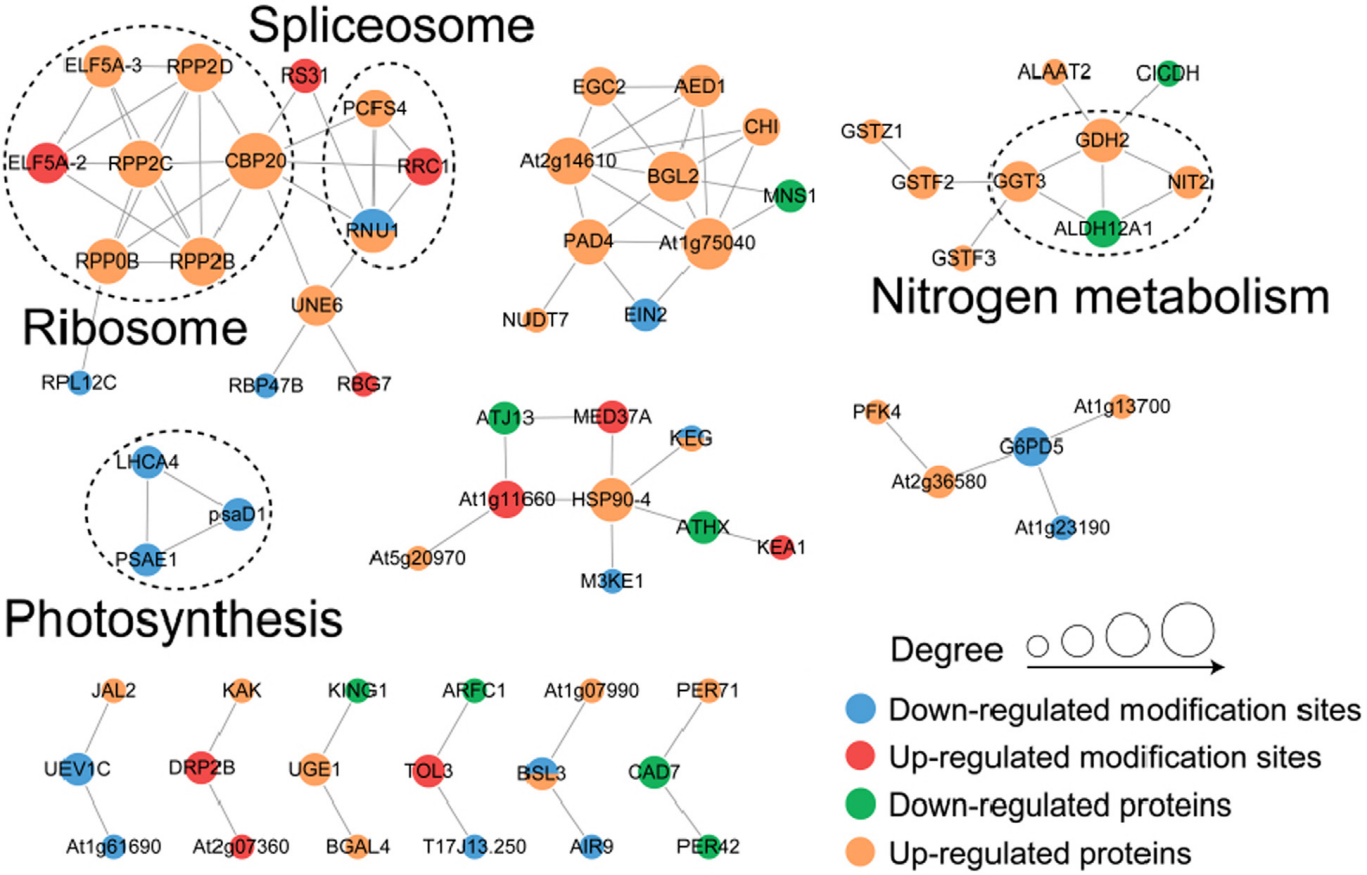
**Interaction networks of the proteome and phosphoproteome.** Node sizes are scaled by median log2 fold change (FC). Edge thicknesses represent the association confidence between two connected nodes and range from 0.7 to 1.0, as determined by String-DB. Highlighted regions of grouped proteins depict the broader process relationships between associating proteins. Nodes with no connections ≥0.7 are not depicted. *Blue*: downregulated modification sites; *Red*: upregulated modification sites; *Green*: downregulated proteins; *Orange*: upregulated proteins. ELF5A-2, ELF5A-3, RPP0B, RPP2B, RPP2C, RPP2D, and CBP20 are ribosomal-related proteins; PCFS4, RRC1, and RNU1 are spliceosome-associated proteins; GGT3, GDH2, NIT2, and ALDH12A1 are proteins related to nitrogen metabolism; LHCA4, PSAE1, and psaD1 are proteins related to photosynthesis.

To determine connections between proteins with changed abundances or phosphorylation, we next built functional association networks for each cluster using STRING-DB (http://string-db.org; Fig. 5) to estimate association confidence between protein nodes. Protein association networks exhibiting significant changes in abundance and phosphorylation status in *vpt1* mutant plants were constructed based on protein–protein interaction, experimental evidence, and coexpression data from the STRING-DB database (Fig. 5). The association networks showed several clusters of interest.

Remarkably, together with our proteomic and phosphoproteomic data, the induced protein abundance and phosphorylation involved in translation and post-transcriptional regulation suggest that vacuolar Pi starvation or increased cytosolic Pi level results in the improvement in protein synthesis *via* integrating ribosome biogenesis, translation, and RNA splicing. The photosynthesis pathway is also affected by VPT1 in Arabidopsis, especially proteins involved in light harvesting in photosystem I.

The proteins involved in nitrogen (N) metabolism including NIT2, glutathione hydrolase 3 (GGT3, At4g29210), glutamate dehydrogenase 2 (GDH2, At5g07440), and delta-1-pyrroline-5-carboxylate dehydrogenase 12A1 (ALDH12A1/P5CDH, At5g62530) were changed and associated. GGT3 catalyzes the first step in catabolism of GSH conjugates in the vacuole and may have an important role in protecting plants from some xenobiotic chemicals (49). *GDH2* is a target of SENSITIVE TO PROTON RHIZOTOXICITY 1 (STOP1) and is a novel aluminum-resistance gene as well as critical for low-oxygen tolerance in Arabidopsis (50, 51). Proline degradation enzyme P5CDH controls reactive oxygen species accumulation and negatively regulates salt-stress tolerance (52). The Arabidopsis response to Pi starvation is closely linked with nitrogen metabolism, reduction and uptake, and during Pi starvation, plants enhanced Pi but reduced nitrate (NO_3_^−^) uptake capacity (53, 54). According to the function, these changed proteins were supposed to improve the stress resistance of *vpt1*.

### Transcript-Level Analysis of DAPs

**Fig. 6:**
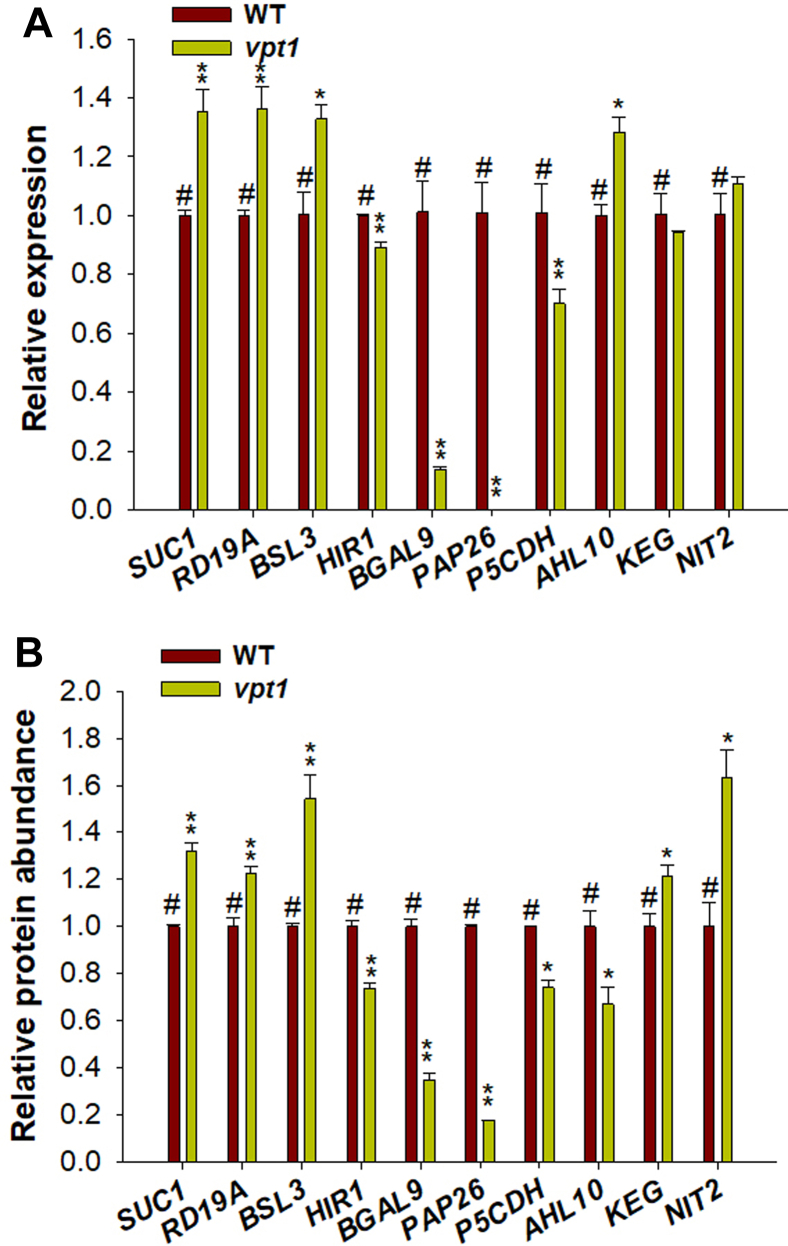
**Transcript-level analysis of typical DAPs.***A*, relative expression of *SUC1*, *RD19A*, *BSL3*, *HIR1*, *BGAL9*, *PAP26*, *P5CDH*, *AHL10*, *KEG*, and *NIT2* in *vpt1* mutant and WT seedlings was detected by RT–quantitative PCR. Three biological replicates were tested for all samples. *ACTIN2* (At3g18780) was used as an internal control, and the relative expression levels were calculated using the 2^−△△Ct^ method. The gene-specific quantitative PCR primers are shown in Supplemental Table S3. *B*, the relative protein abundance was analyzed using the normalized intensity of SUC1, RD19A, BSL3, HIR1, BGAL9, PAP26, P5CDH, AHL10, KEG, and NIT2 in Supplemental Table S5. DAP, differential abundance protein.

To clarify whether these significantly changed proteins in the proteome are controlled at the transcriptional level, we examined some typical proteins of interest at the transcriptional level by RT–qPCR. The analysis revealed that the expression of *SUC1*, *RD19A*, and *BSL3* was elevated along with an increase in the abundance of the proteins in the *vpt1* mutant compared with WT. The transcription expression of *HIR1*, *BGAL9*, *PAP26*, and *P5CDH* decreased accompanied by a decrease in protein abundance (Fig. 6*A*). It suggested that these protein changes may be caused by the transcription-level changes.

Notably, the protein abundance changes in AHL10, NIT2, and KEG were inconsistent with their transcription changes. The transcription expression of *AHL10* increased, but protein abundance decreased (Fig. 6*B*). And the phosphoproteome data showed that the phosphorylation levels of AHL10 were elevated (Fig. 3*D*). So, it is speculated that AHL10 would be degradated after phosphorylation modification. The change of the transcription expression of *NIT2* and *KEG* was not significant, but the protein abundance was significantly higher in the mutant than in the WT, with a decreased phosphorylation level (Figs. 6 and 3*D*), indicating that the NIT2 and KEG protein levels would be regulated by post-translational modification.

## Conclusion

**Fig. 7:**
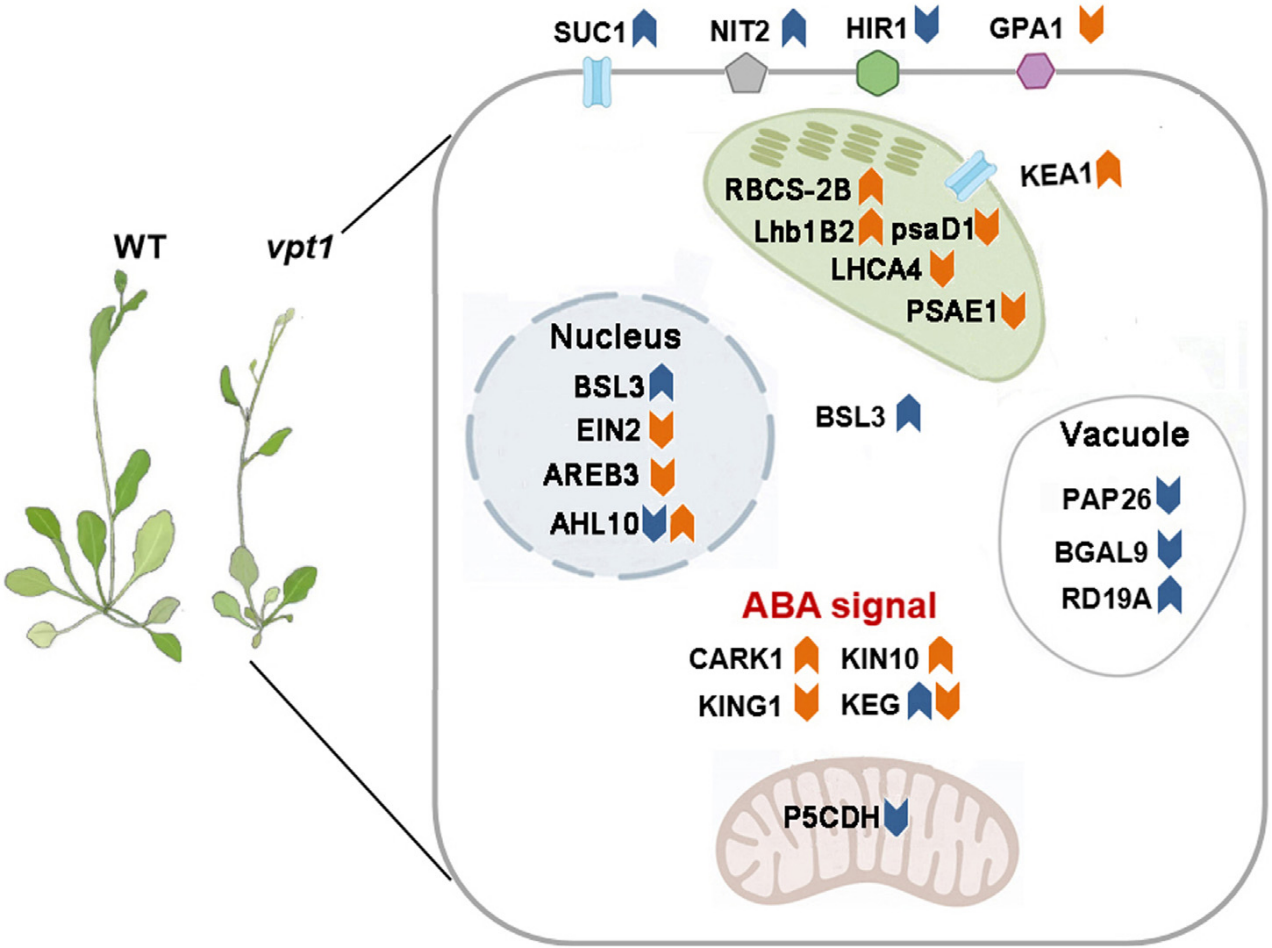
**Summary of observed changes in protein abundance and protein phosphorylation in *vpt1* mutant.***Blue arrows* represent differential proteins in proteomic, and *orange arrows* represent differential proteins in phosphoromic. AHL10, AT-HOOK MOTIF NUCLEAR LOCALIZED PROTEIN 10; ALDH12A1/P5CDH, delta-1-pyrroline-5-carboxylate dehydrogenase 12A1; AREB3, ABSCISIC ACID-INSENSITIVE 5-like protein; ARK1, cytosolic ABA receptor kinase1; BGAL9, beta-galactosidase9; BSL3, BRI1 SUPPRESSOR 1 (BSU1)-LIKE 3; EIN2, ETHYLENE INSENSITIVE2; GPA1, guanine nucleotide-binding protein alpha-1 subunit; HIR1, hypersensitive-induced response protein1; KEA1, K^+^ efflux antiporter; KEG, KEEP ON GOING; KIN10, SnRK1 catalytic subunit alpha; KING1, SnRK1 regulatory subunit gamma-1; Lhb1B2, chlorophyll a–b binding protein; LHCA4, chlorophyll a–b binding protein 4; NIT2, nitrilase 2; PAP26, purple acid phosphatase26; psaD1, photosystem I reaction center subunit II-1; PSAE1, photosystem I reaction center subunit IV A; RBCS-2B, ribulose bisphosphate carboxylase small chain 2B; RD19A, responsive to dethydration19A; SUC1, sucrose transport protein.

In summary, we have performed in-depth protein abundance and phosphorylation state profiling of *vpt1* mutant plants. The findings allow us to propose a working model for system vacuolar Pi starvation or increased cytosolic Pi level signal network (Fig. 7). The aforementioned highlighted observations suggest that these datasets will enable the discovery of novel proteins, including protein kinases and TFs, and biological processes dependent on phosphate signaling. The function of many proteins such as E3 ligase KEG, protein kinase SnRK1, and TF AREB3 was revealed. The interaction between phosphate signal transduction, photosynthesis, and plant hormone especially ABA deserves to be paid more attention. This study could provide a cue that how the system phosphate signal effect photosynthesis, carbohydrate metabolic process, and plant hormone. Above all, our study illuminates several new aspects of the phosphate starvation or toxicity response for further investigation and potential crop improvement.

## Data Availability

The datasets analyzed in this article are available online. Mass spectrometry proteome and phosphoproteome data have been deposited on the ProteomeXchange Consortium *via* the iProX partner repository. Project name: The proteome and phosphoproteome uncovers candidate proteins associated with vacuolar phosphate signal multipled by VPT1 in Arabidopsis. Dataset identifier: PXD040698. The data access connection in ProteomeXchange is http://proteomecentral.proteomexchange.org/cgi/GetDataset?ID=PXD040698. The annotated spectra for phosphopeptides and single-unique-peptide-identified proteins can be viewed using the following URL: https://msviewer.ucsf.edu/prospector/cgi-bin/mssearch.cgi?report_title=MS-Viewer&search_key=lsbngdwgy8&search_name=msviewer. The search key is lsbngdwgy8 on MS-Viewer.

## Supplemental data

This article contains supplemental data.

## Conflict of interest

The authors declare no competing interests.

## Figures and Tables

**Supplemental Figure S3:**
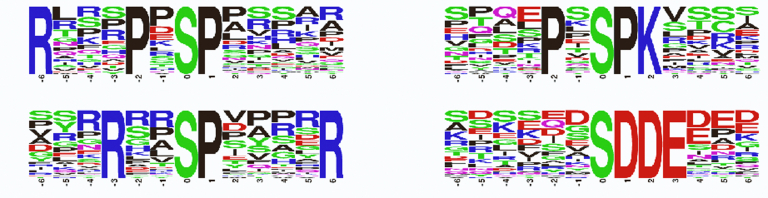
**Motif analysis of significantly enriched phosphopeptides in the phosphoproteomics data**.

## Supplementary Data

Supplemental Table S1 MS identified information of proteome.

Supplemental Table S2 MS identified information of phosphoproteome.

Supplemental Table S3 Primers used for RT-qPCR.

Supplemental Table S4 All identified and quantified proteins in proteome.

Supplemental Table S5 Significantly changing abundance proteins in proteome.

Supplemental Table S6 All identified and quantified phosphoproteins.

Supplemental Table S7 Significantly changing phosphoproteins.

Supplemental Table S8 Overlap proteins between proteome and phosphoproteome.

Supplemental Table S9 GO enrichment of significantly changing abundance proteins.

Supplemental Table S10 GO enrichment of significantly changing phosphoproteins.

Supplemental Table S11 The key spectums of significantly changing proteins in Fig. 7.
