# Supplementary material for: The Proteome and Phosphoproteome Uncovers Candidate Proteins Associated With Vacuolar Phosphate Signal Multipled by Vacuolar Phosphate Transporter 1 (VPT1) in Arabidopsis
Source: Mol Cell Proteomics. 2023 Apr 18;22(6):100549. doi: 10.1016/j.mcpro.2023.100549 (PMC10209694; doi:10.1016/j.mcpro.2023.100549)

Raw file	Scan number	Mass analyzer	Score	m/z	Proteins
F8065TQ_12	9859	FTMS	134.13	1211.605	A0A2H1ZEI5;A0A178VL84;Q9LJX4;Q9ZW07;Q9ZW06;Q9ZW02

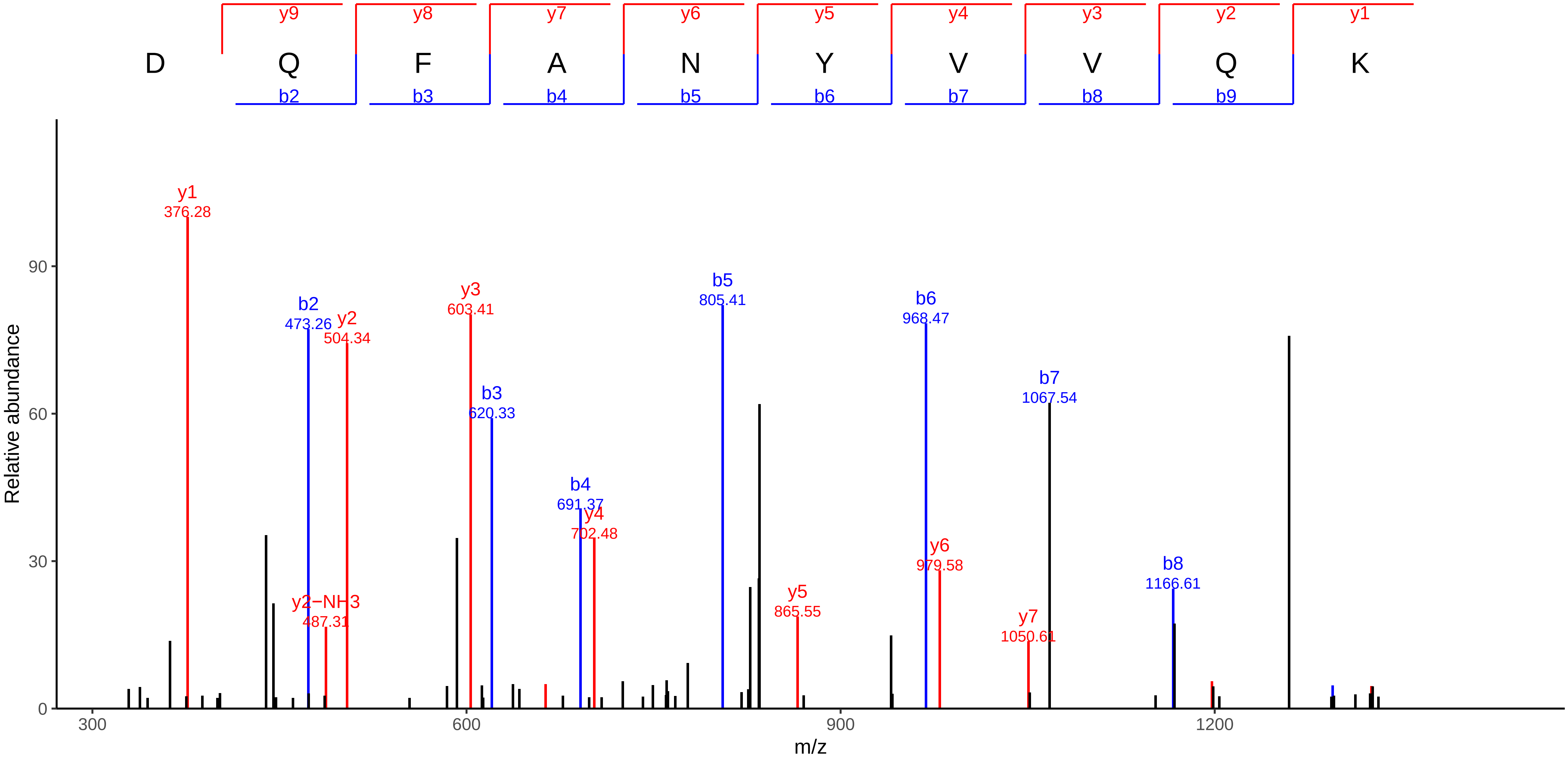

Raw file	Scan number	Mass analyzer	Score	m/z	Proteins
F8065TQ_10	12353	FTMS	135.72	544.6023	Q8L7U5;F4HW26;Q9SJF0;F4IFQ0;Q9SHS7

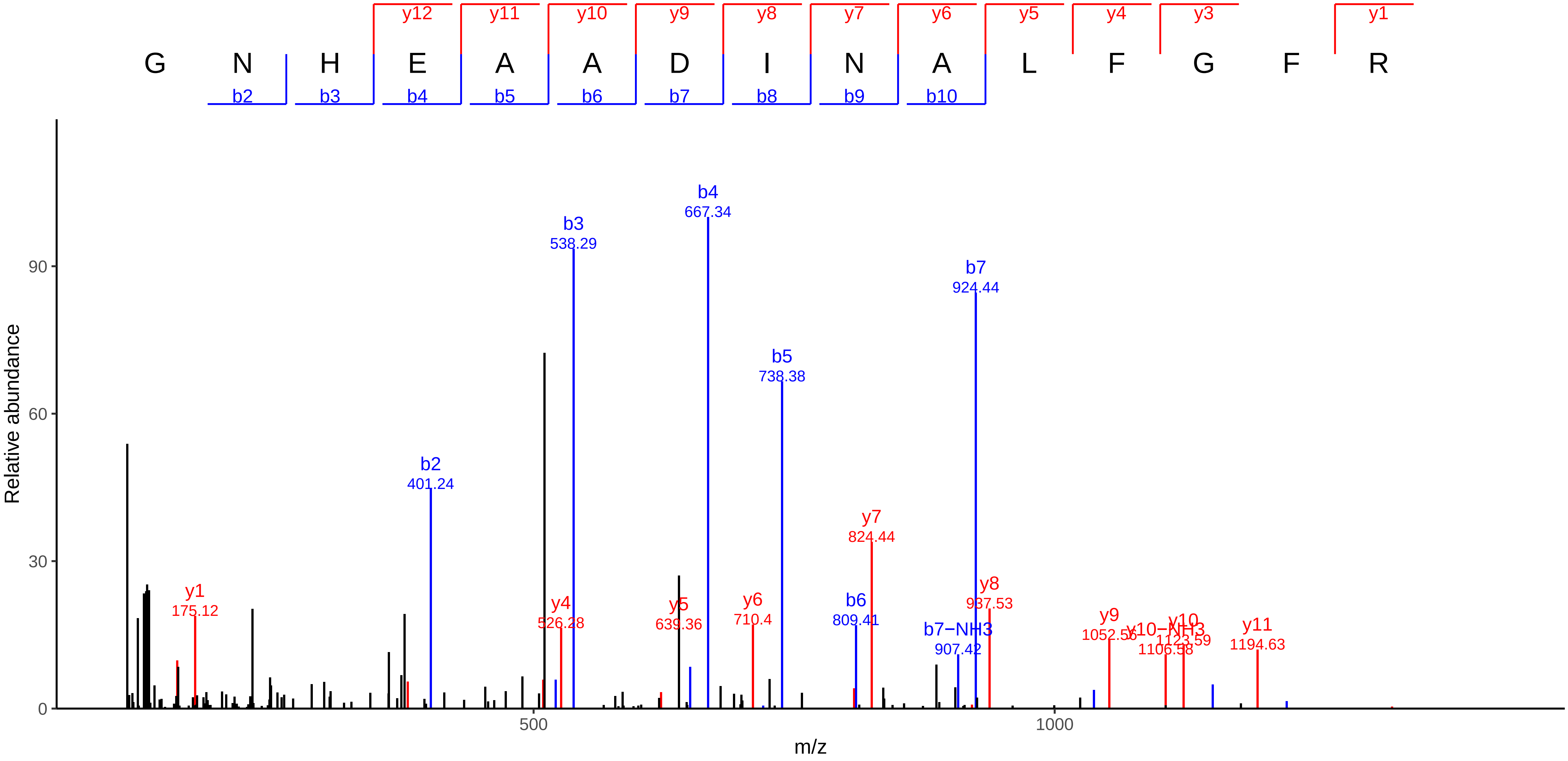

Raw file	Scan number	Mass analyzer	Score	m/z	Proteins
F8065TQ_18	10728	FTMS	133.48	837.4194	F4IUQ7;Q9SCV3

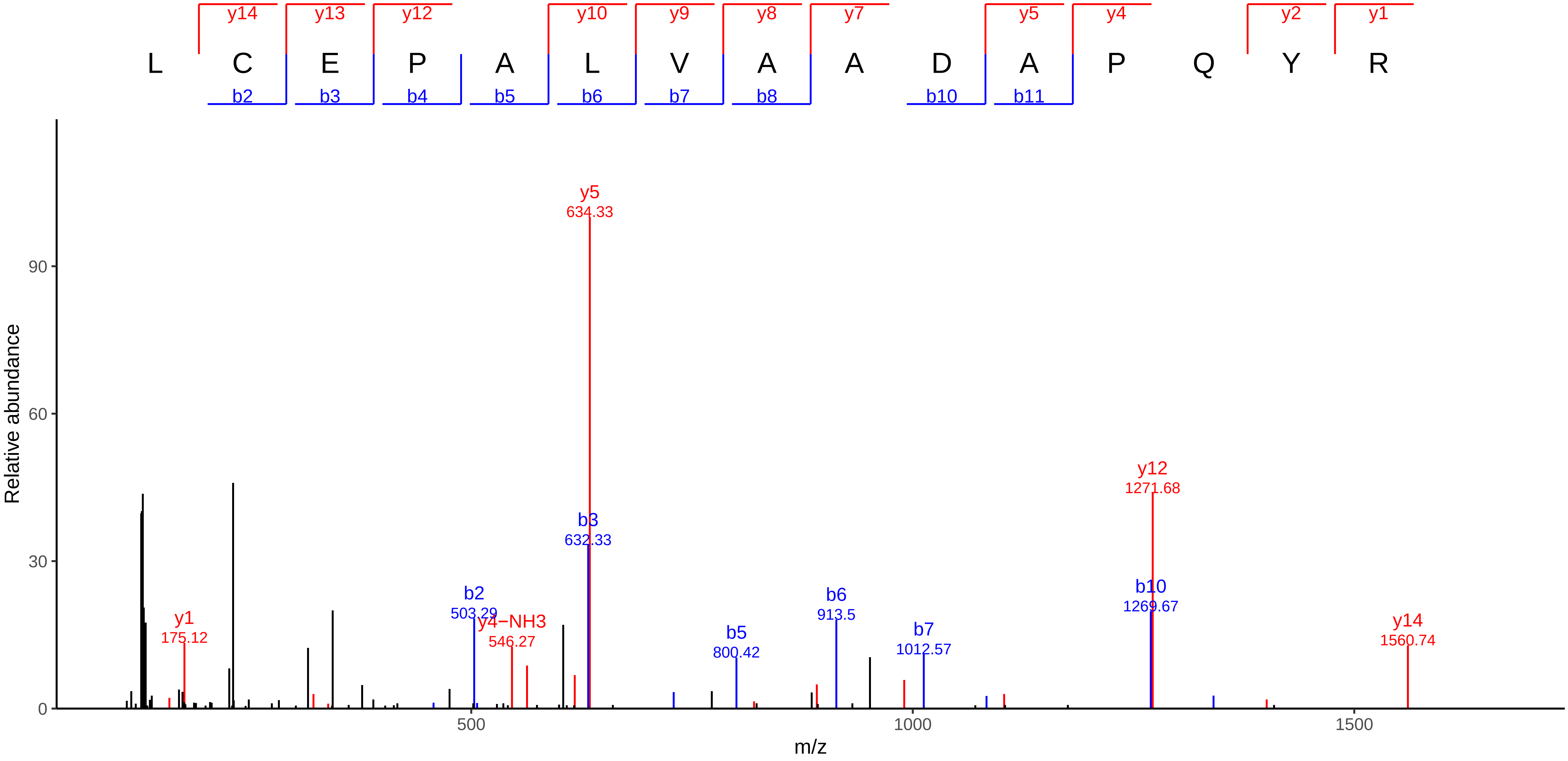

Raw file	Scan number	Mass analyzer	Score	m/z	Proteins
F8065TQ_7	12276	FTMS	68.224	480.7611	F4K3Z6;Q9FY48

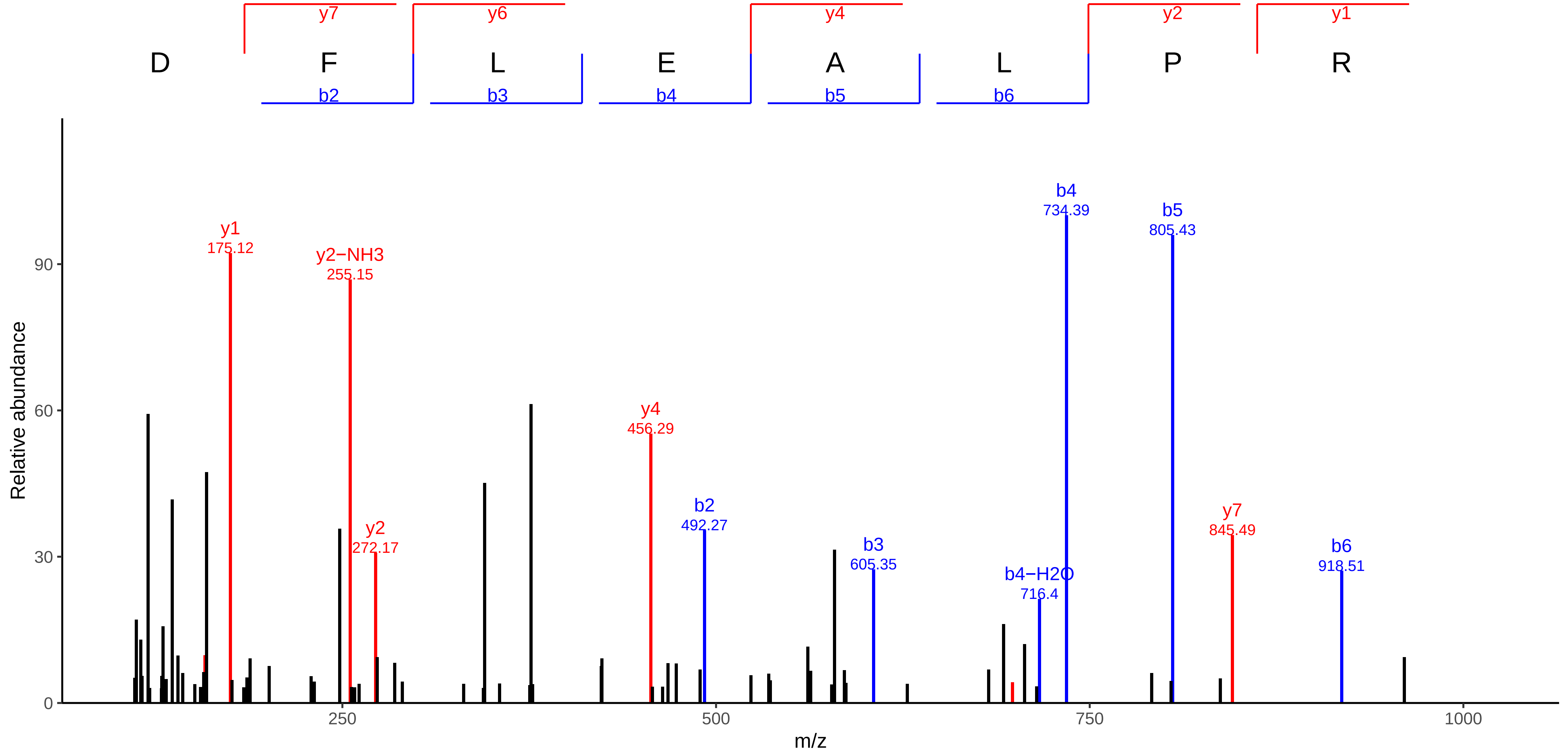

Raw file	Scan number	Mass analyzer	Score	m/z	Proteins
F8065TQ_12	3350	FTMS	78.69	707.3339	O22812

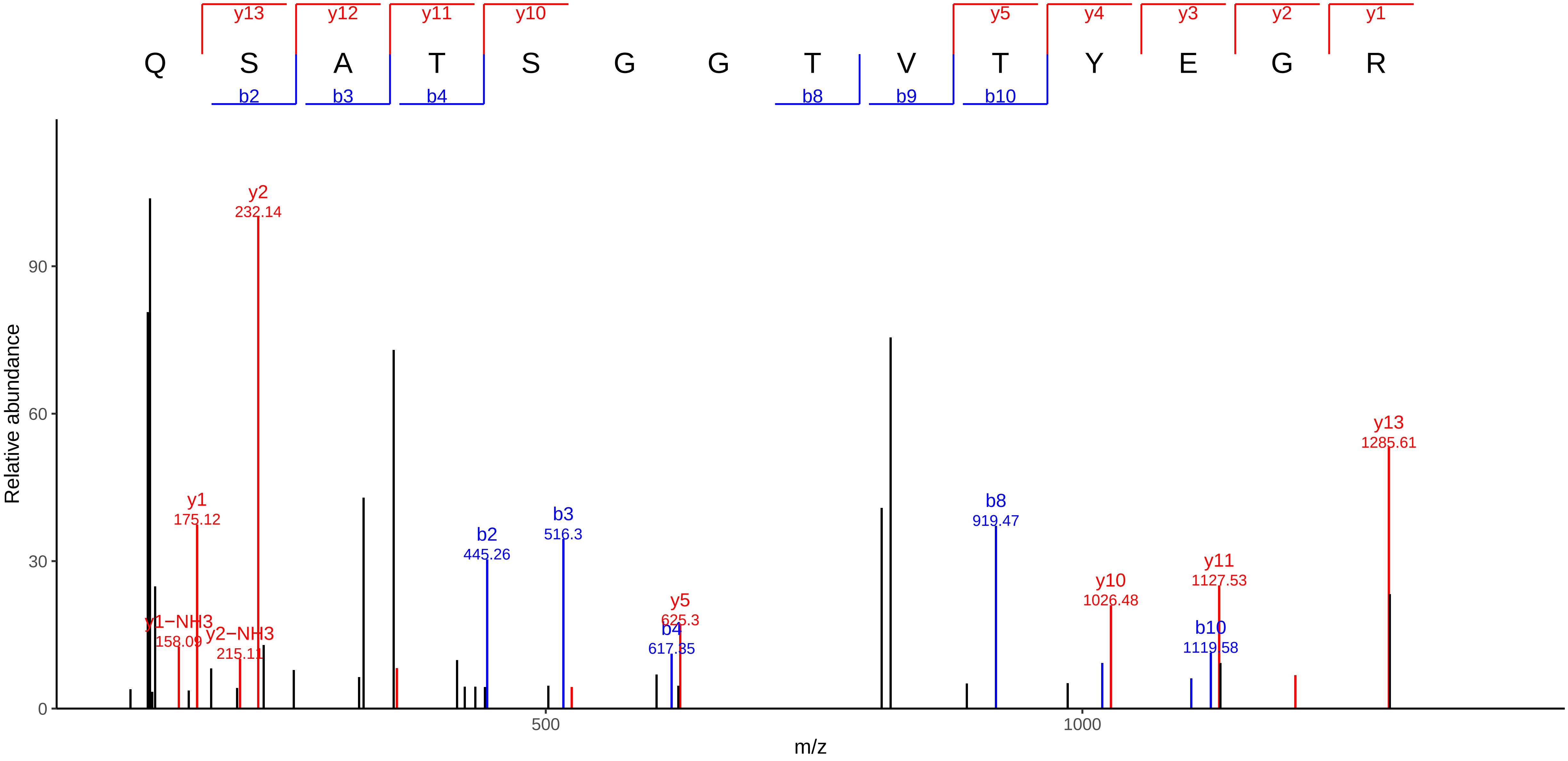

Raw file	Scan number	Mass analyzer	Score	m/z	Proteins
F8065TQ_15	15643	FTMS	132.25	919.4778	P32962

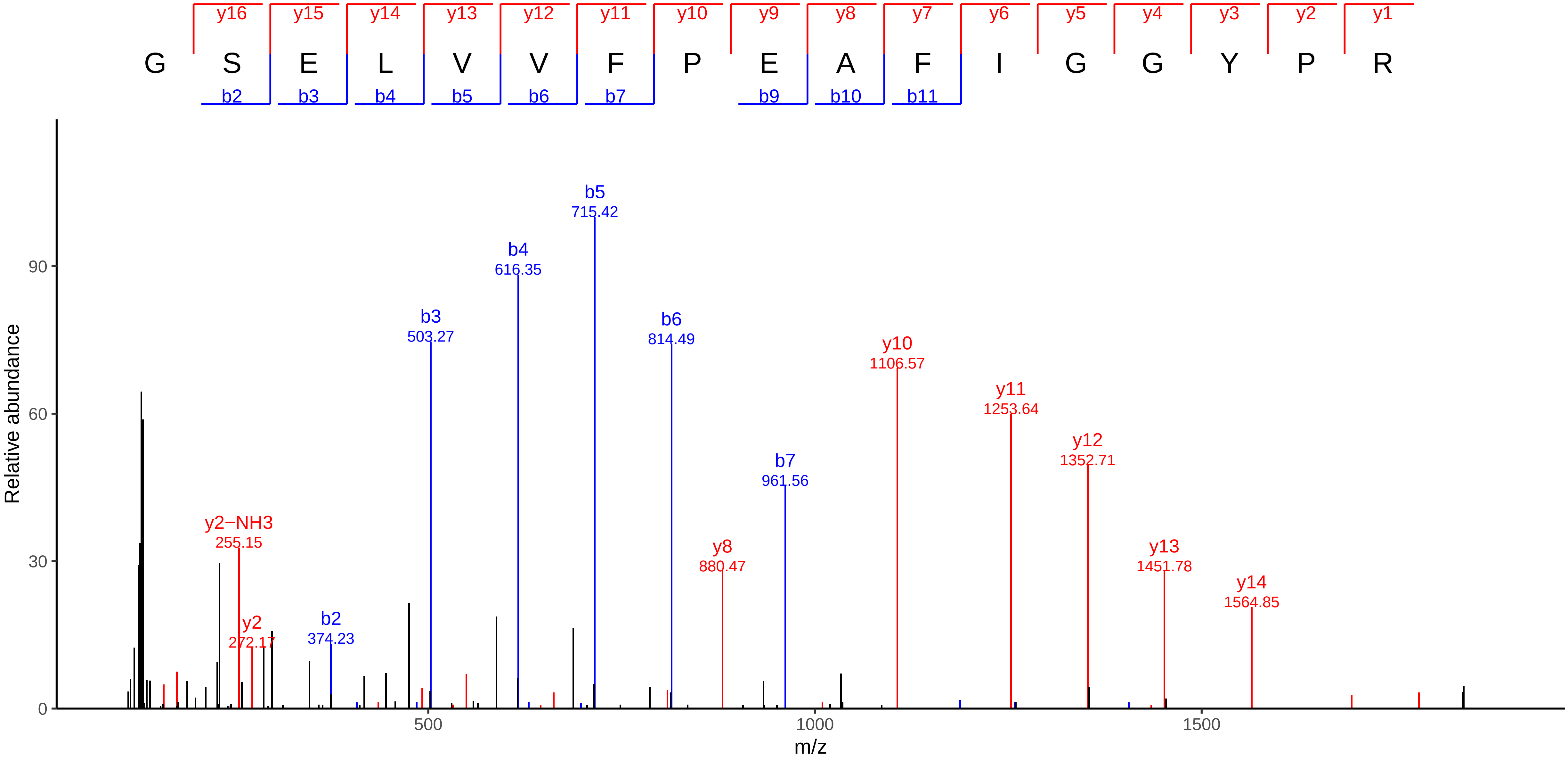

Raw file	Scan number	Mass analyzer	Score	m/z	Proteins
F8065TQ_5	9007	FTMS	102.08	844.8954	P43296

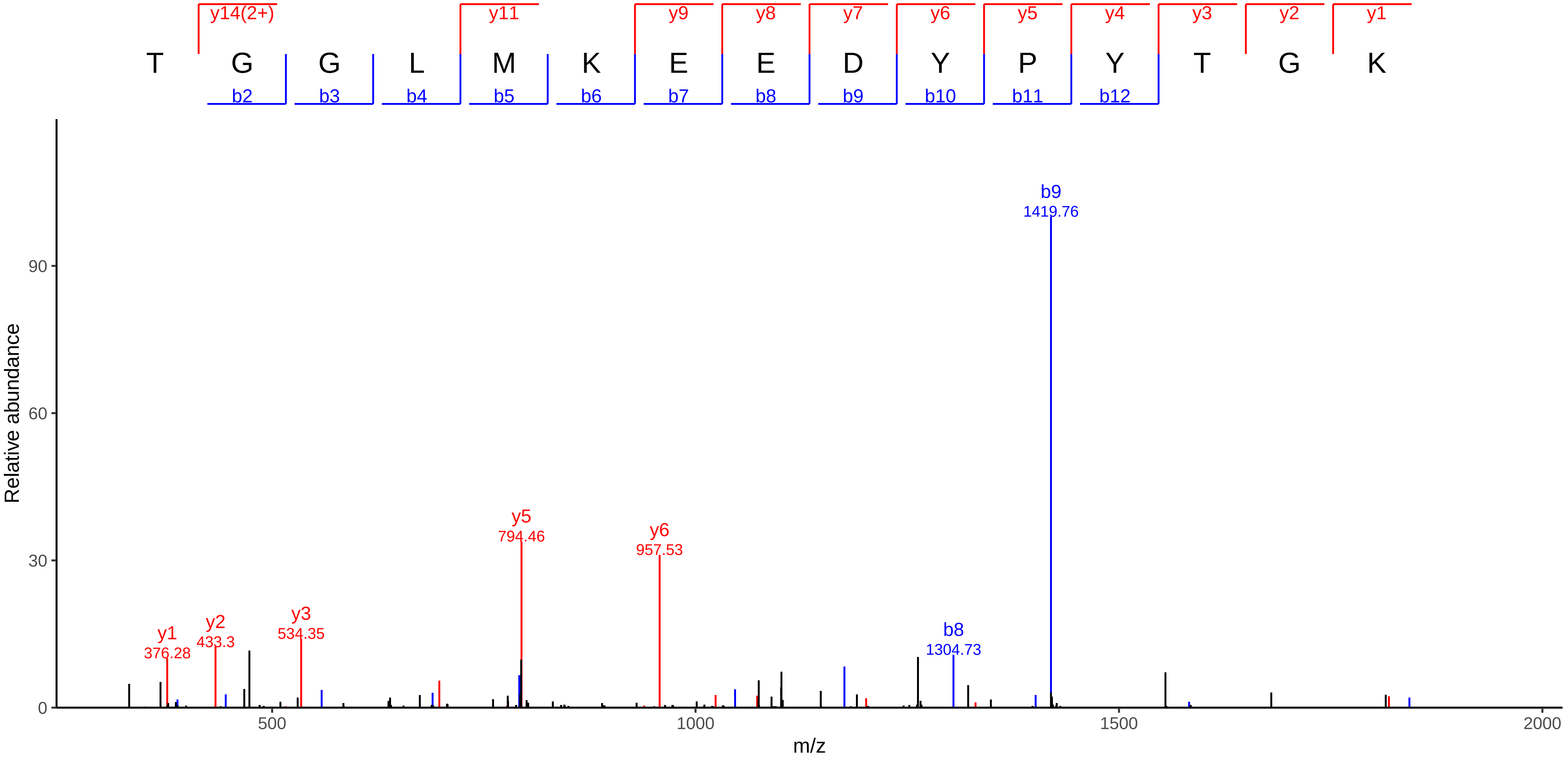

Raw file	Scan number	Mass analyzer	Score	m/z	Proteins
F8065TQ_17	7068	FTMS	161.91	995.9663	Q949Y3

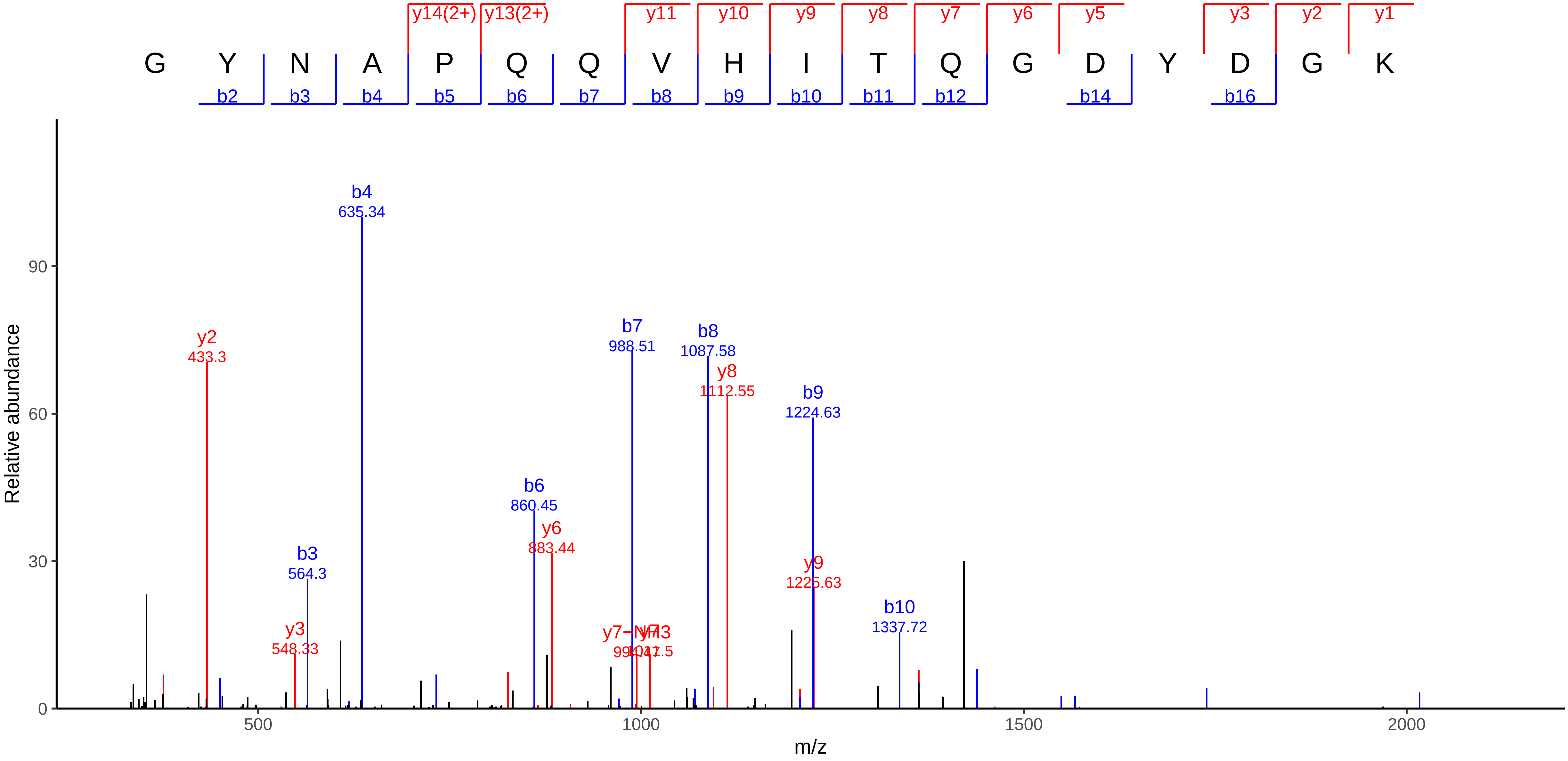

Raw file	Scan number	Mass analyzer	Score	m/z	Proteins
F8065TQ_7	15627	FTMS	149.96	1223.649	Q39241

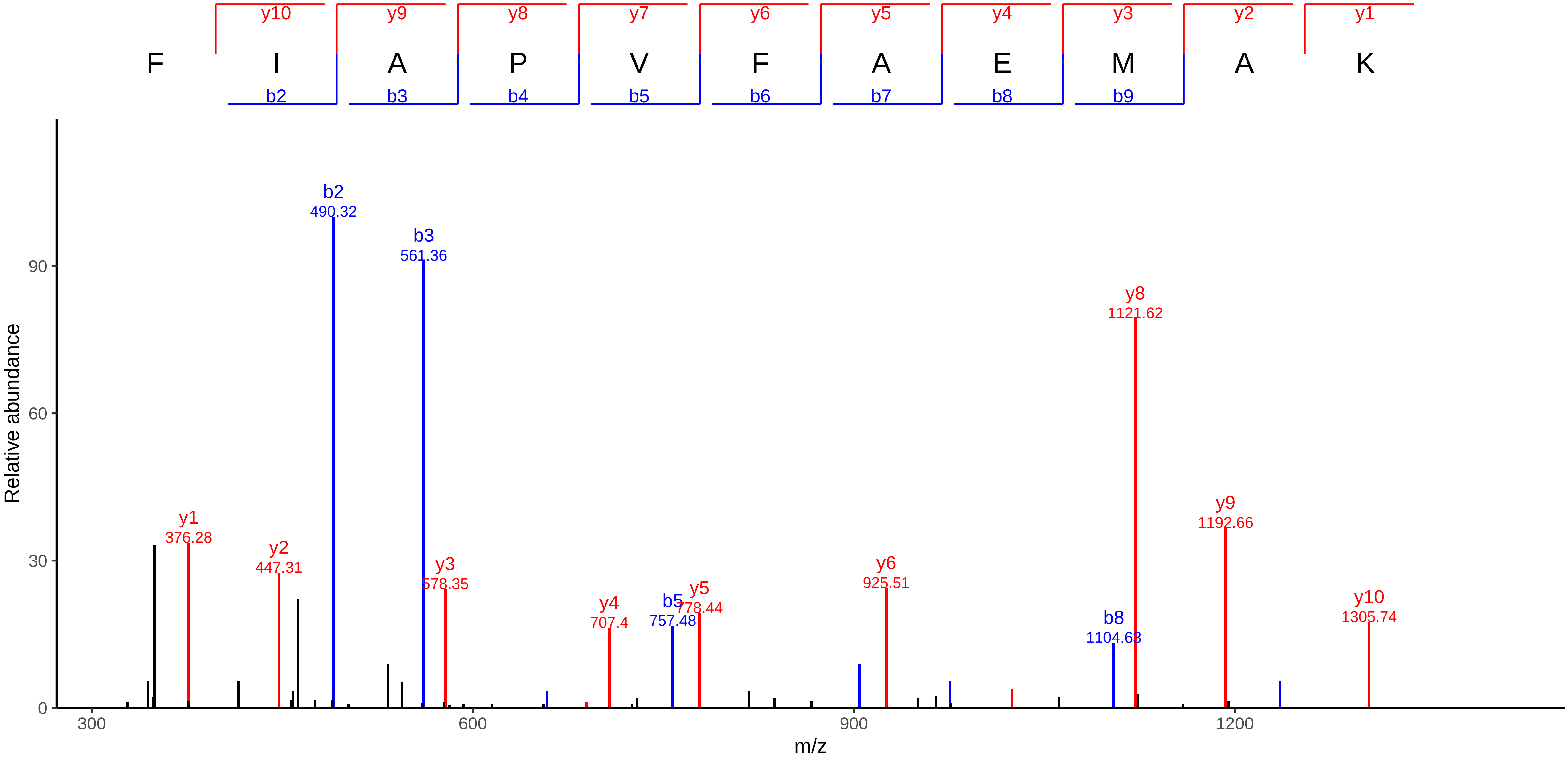

Raw file	Scan number	Mass analyzer	Score	m/z	Proteins
F8065TQ_9	2796	FTMS	150.36	470.7516	Q42404

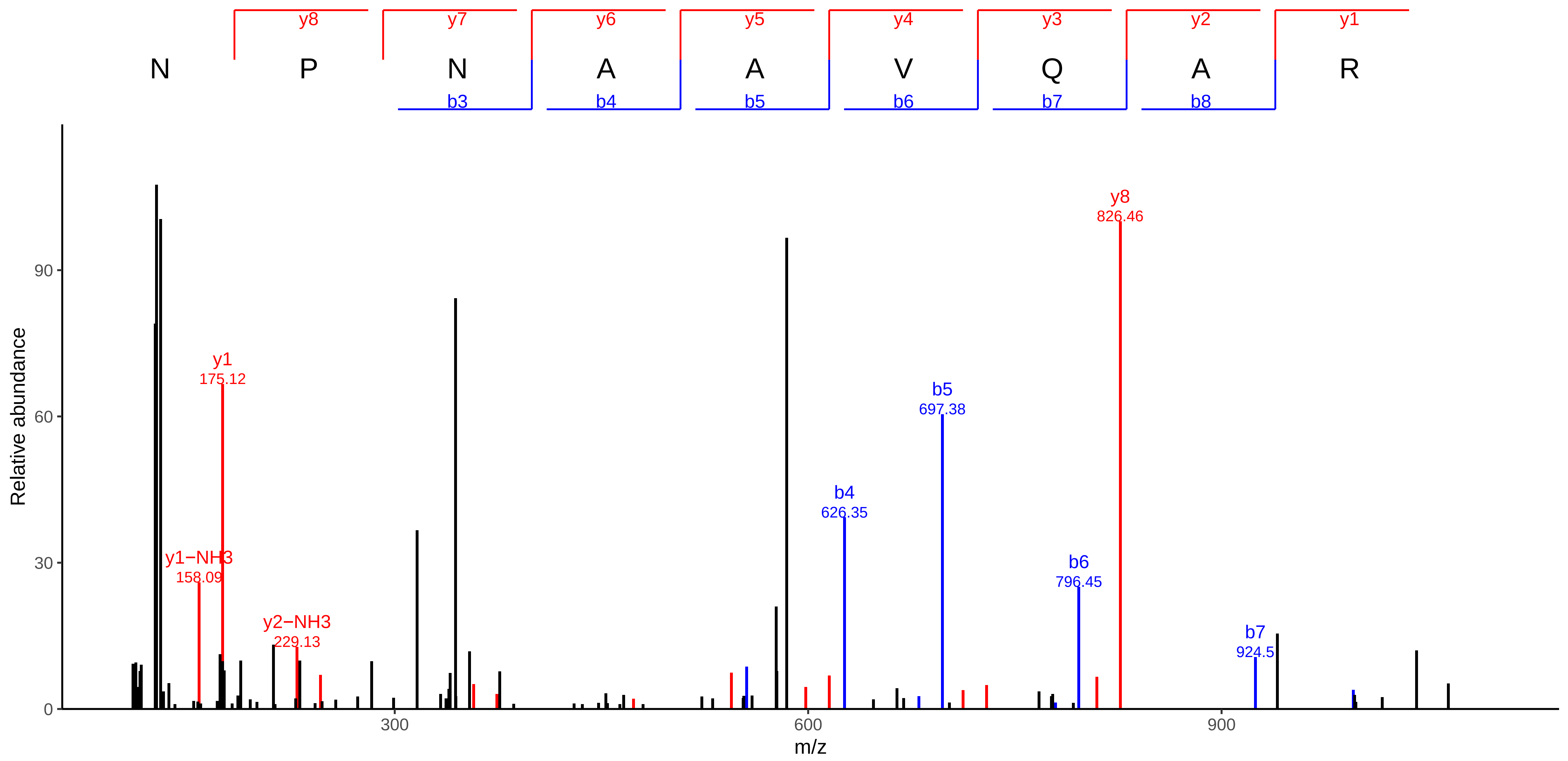

Raw file	Scan number	Mass analyzer	Score	m/z	Proteins
F8065TPST_1	26585	FTMS	69.272	752.9758	A0A2H1ZEI5;A0A178VL84;Q9LJX4

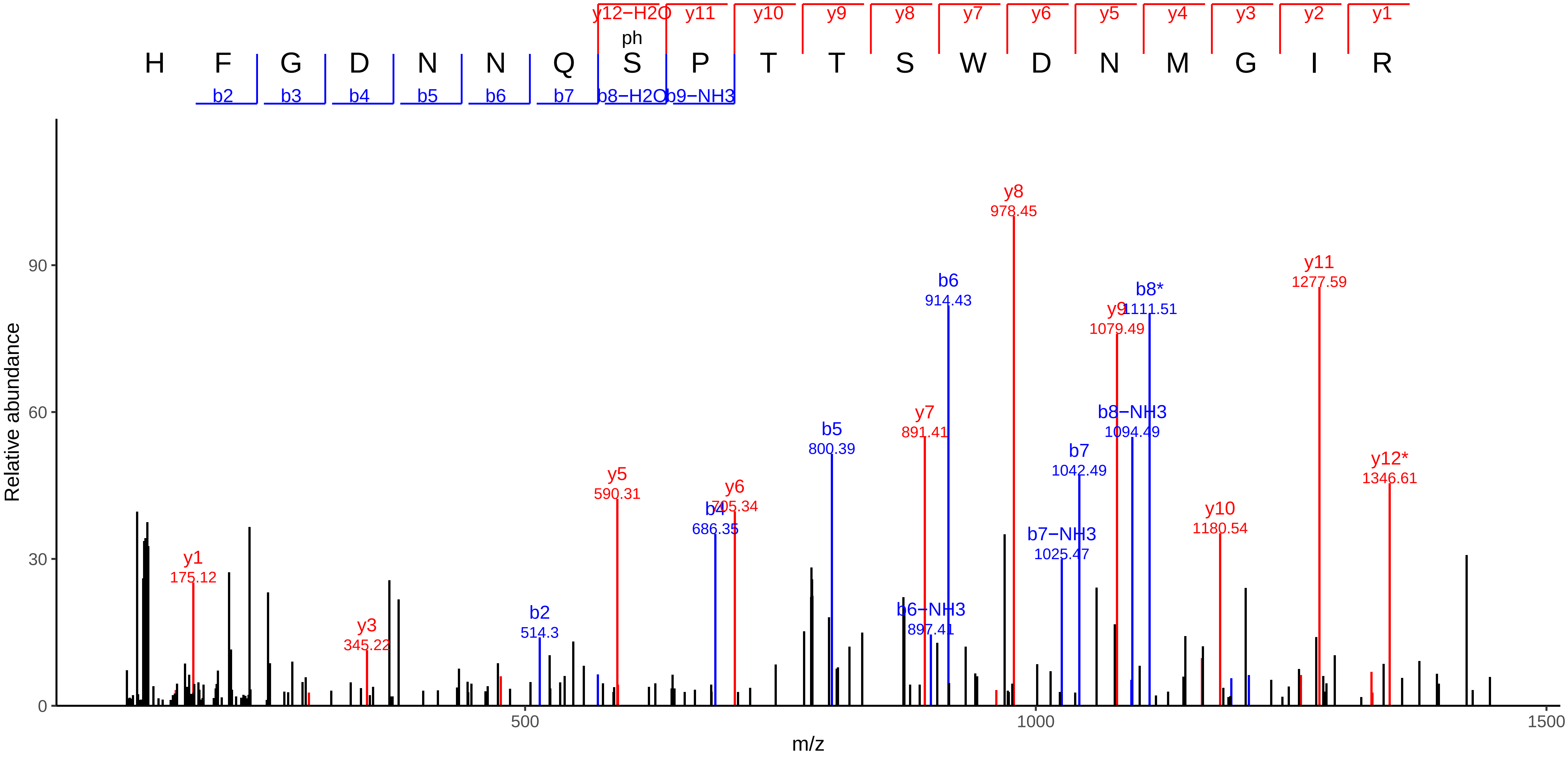

Raw file	Scan number	Mass analyzer	Score	m/z	Proteins
F8065TPST_3	34431	FTMS	132.13	679.0853	F4IFQ0;Q9SHS7

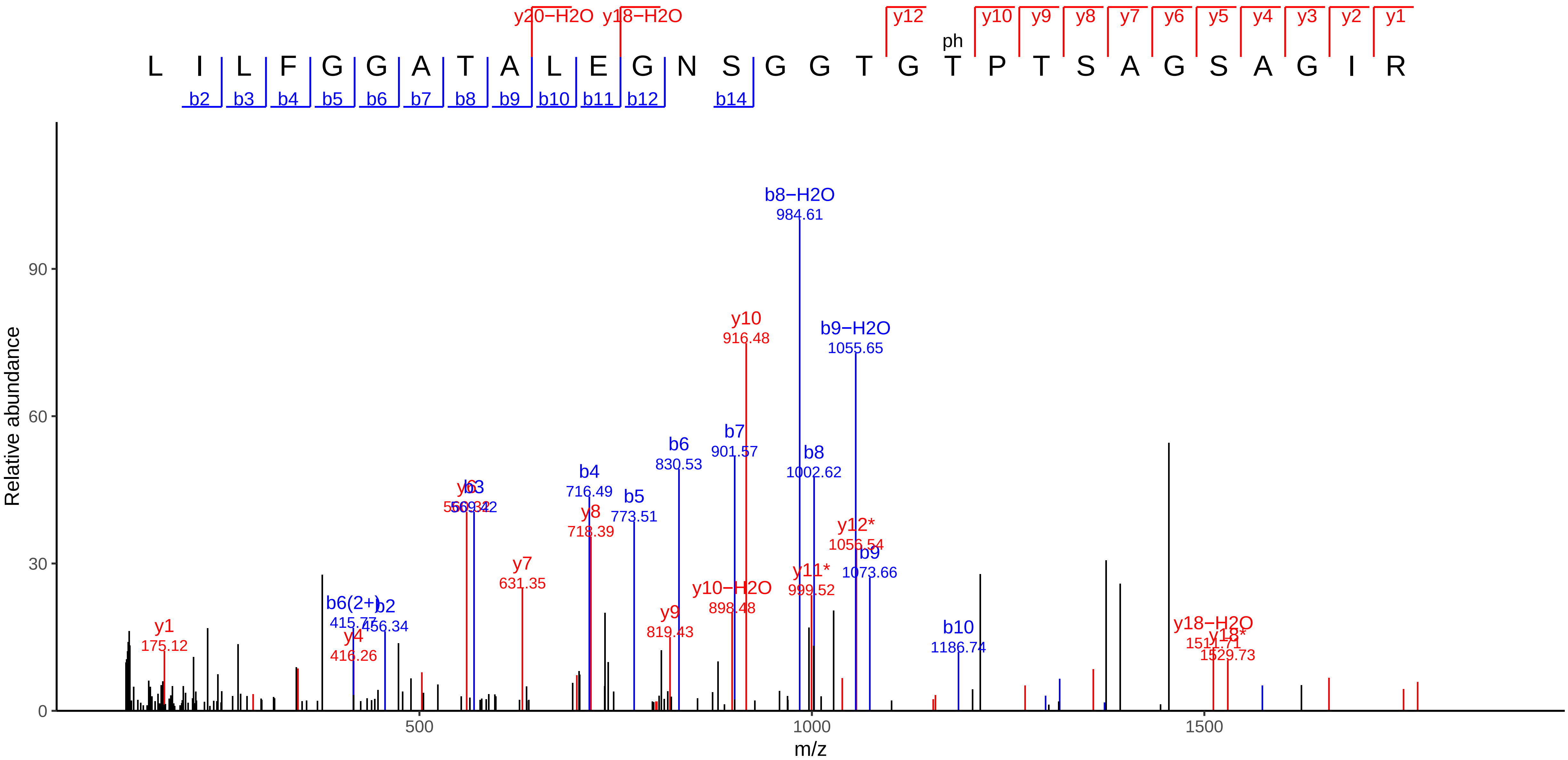

Raw file	Scan number	Mass analyzer	Score	m/z	Proteins
F8065TPST_6	23191	FTMS	98.654	647.2974	F4IFQ0;Q9SHS7

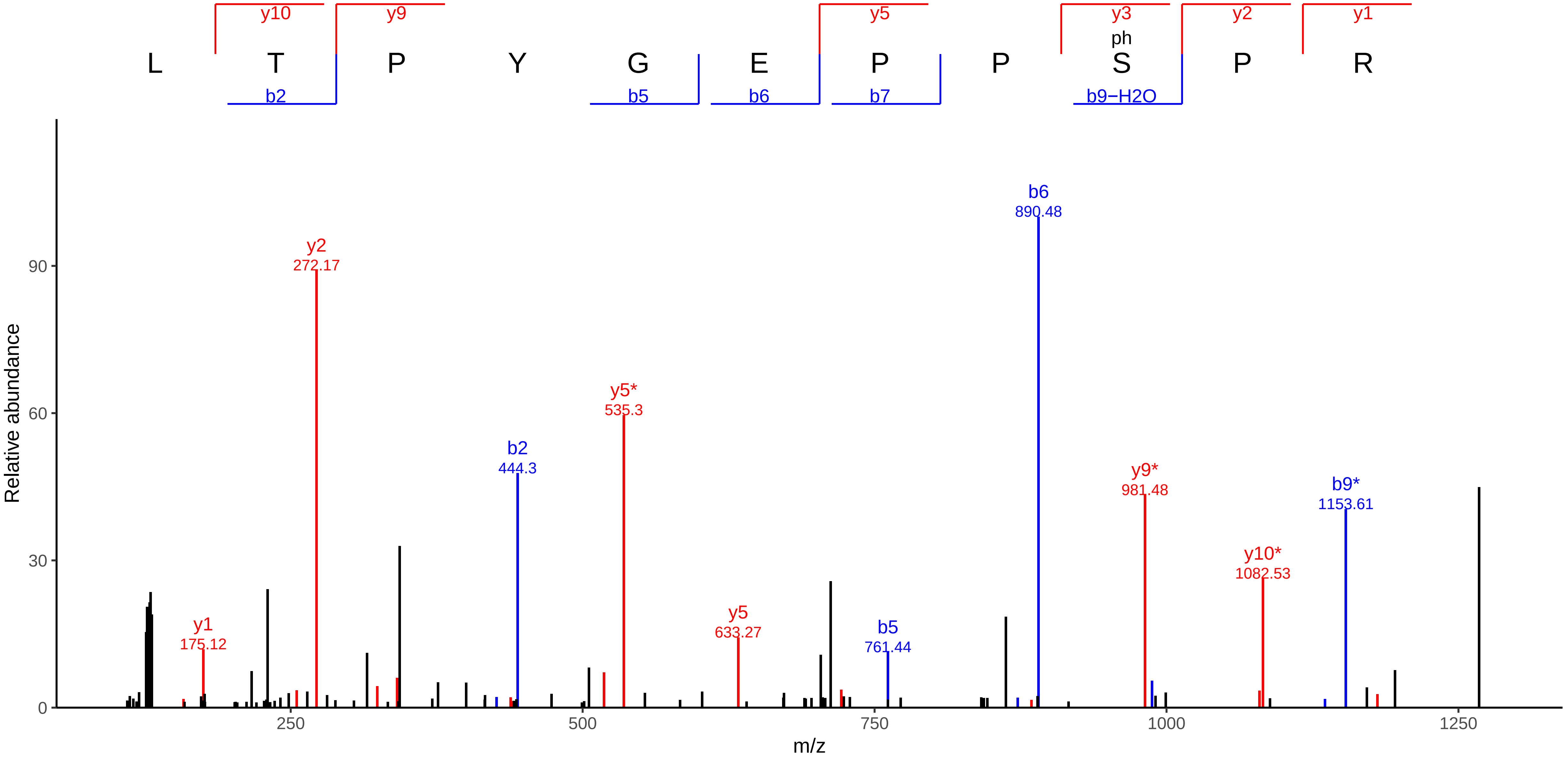

Raw file	Scan number	Mass analyzer	Score	m/z	Proteins
F8065TPST_2	29371	FTMS	193.71	1058	F4IFQ0;Q9SHS7

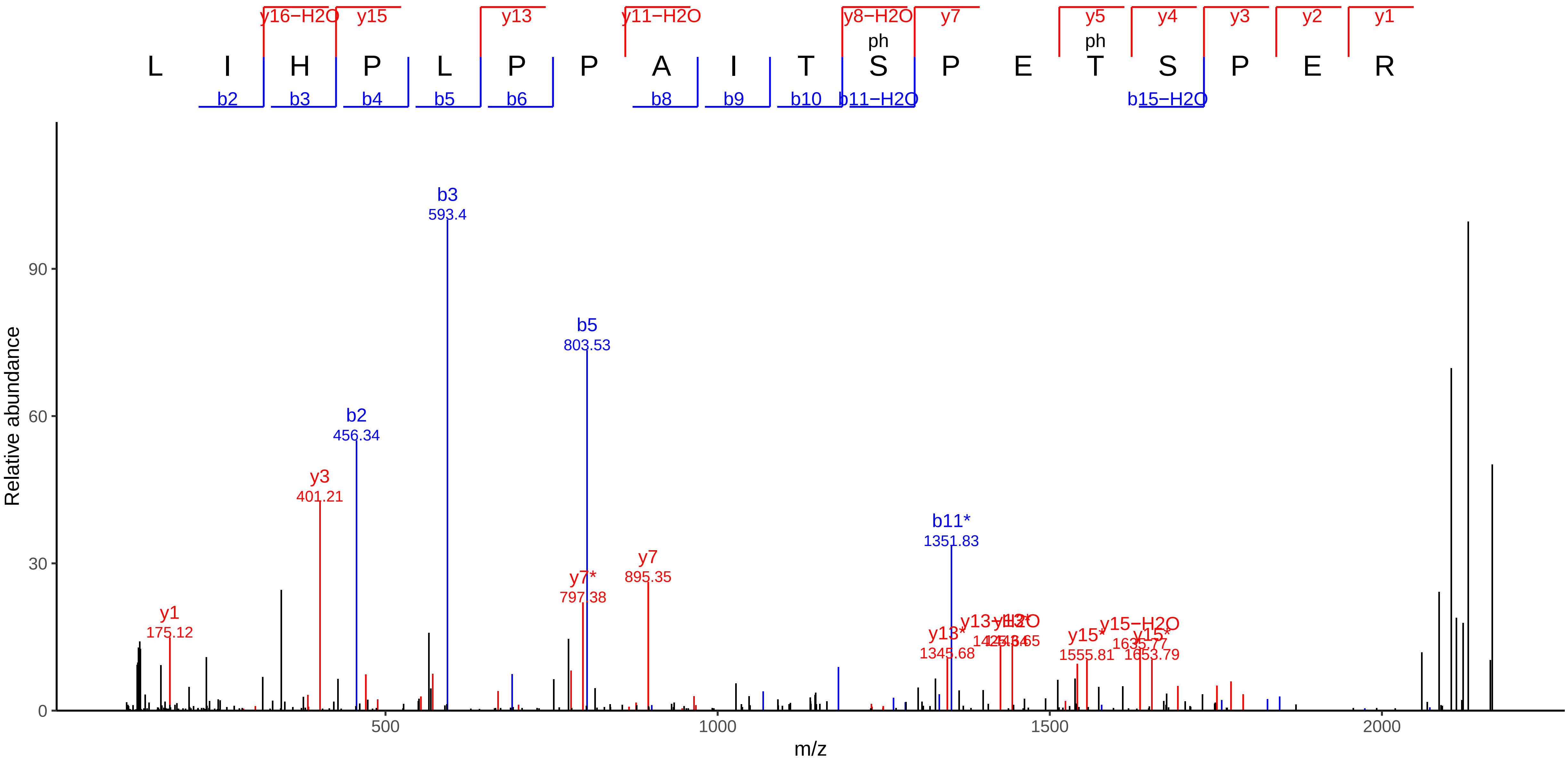

Raw file	Scan number	Mass analyzer	Score	m/z	Proteins
F8065TPST_1	20723	FTMS	127.35	700.0037	F4K3Z6;Q9FY48

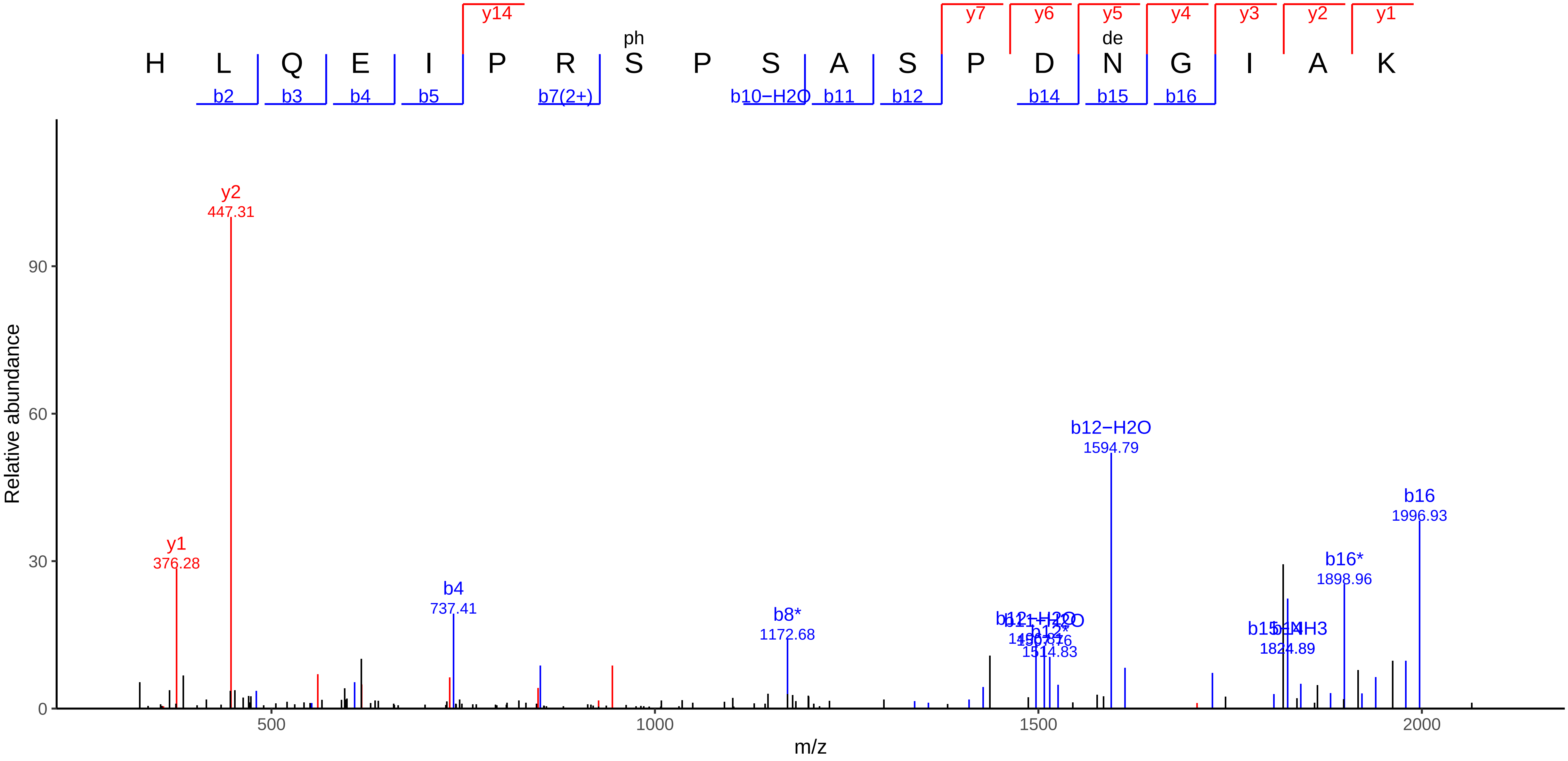

Raw file	Scan number	Mass analyzer	Score	m/z	Proteins
F8065TPST_1	24418	FTMS	149.95	562.6113	O22812

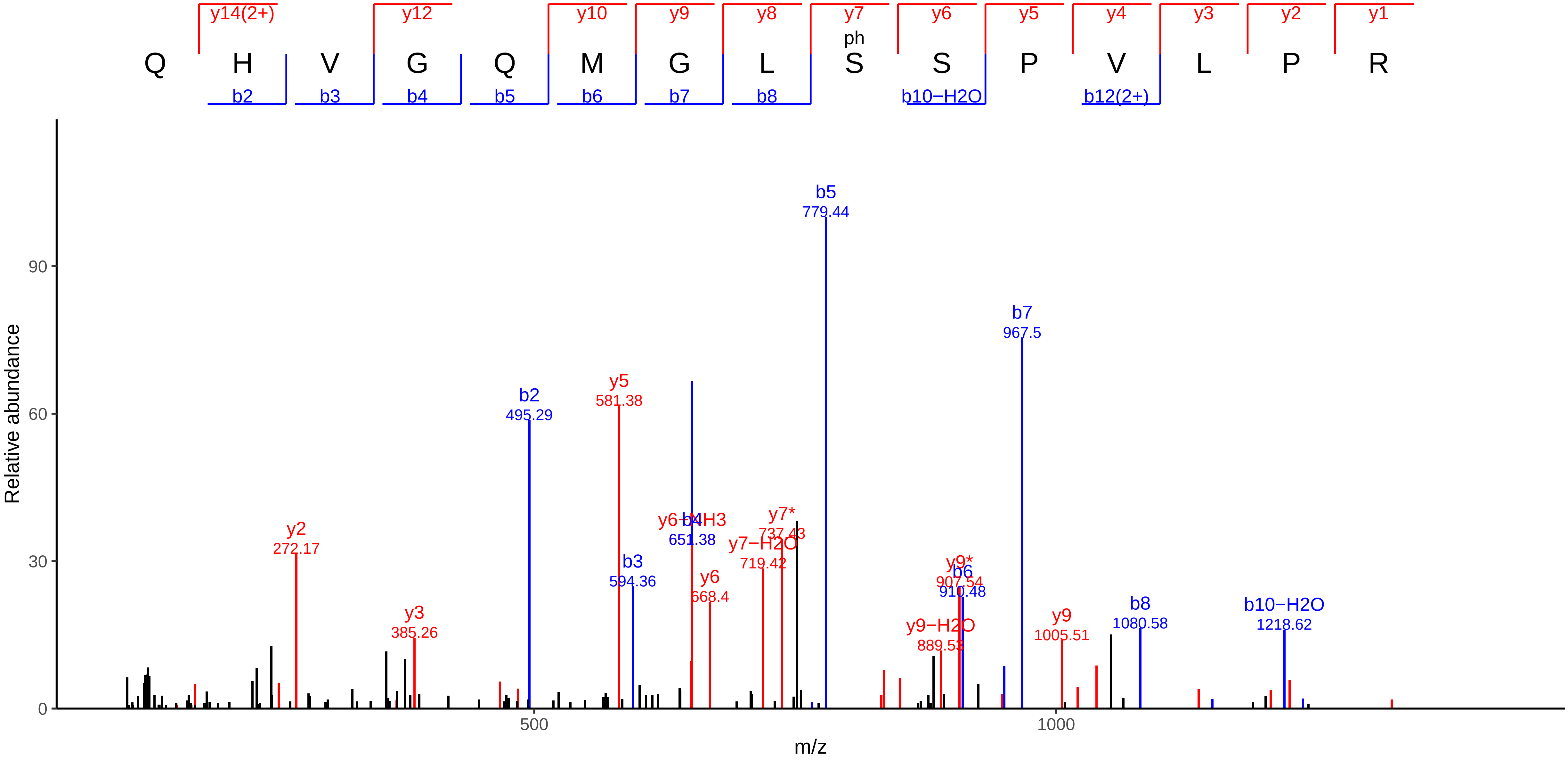

Raw file	Scan number	Mass analyzer	Score	m/z	Proteins
F8065TPST_5	14603	FTMS	44.98	396.176	Q39241

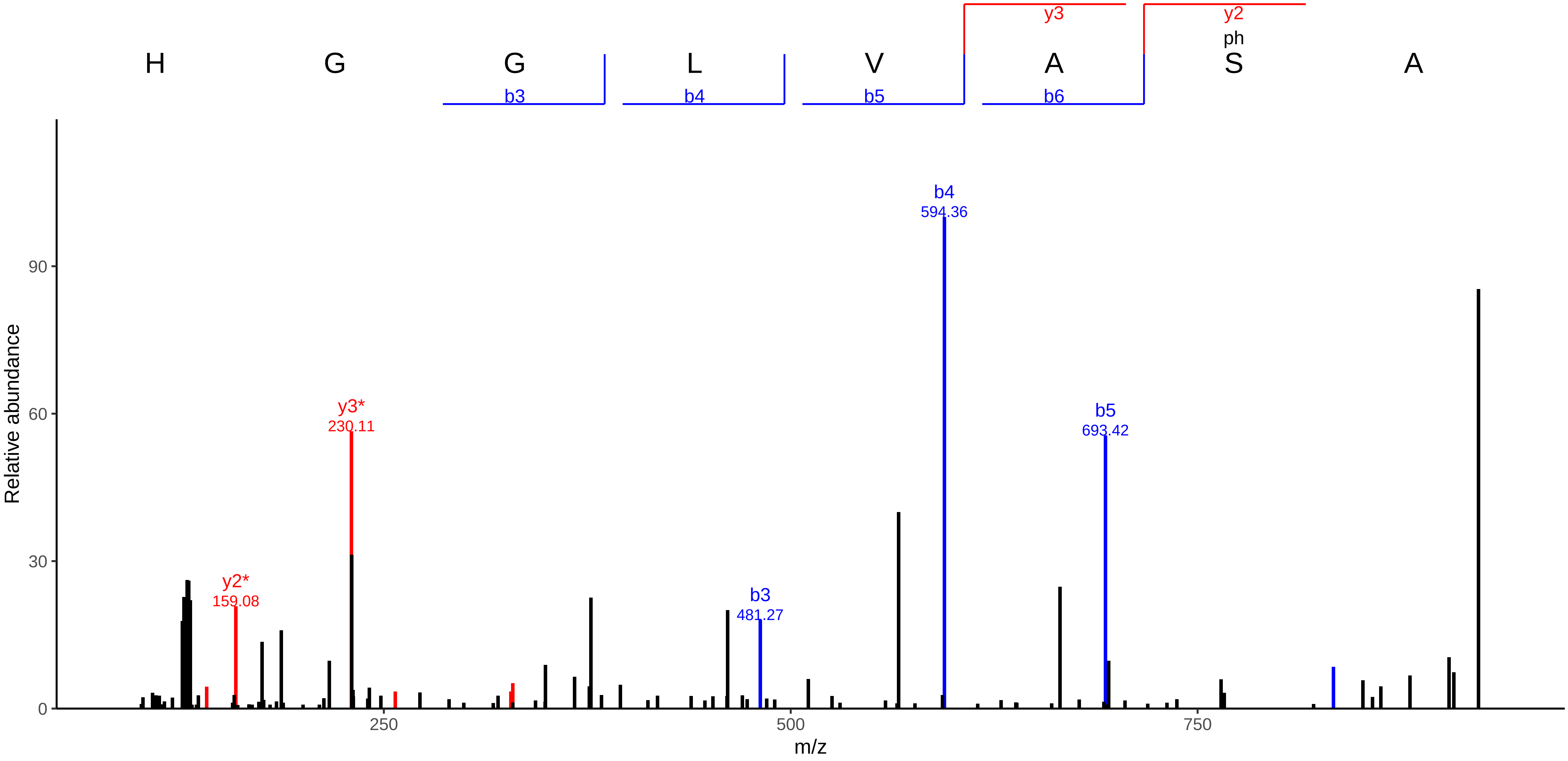

Raw file	Scan number	Mass analyzer	Score	m/z	Proteins
F8065TPST_4	7615	FTMS	102.33	631.2879	Q42404

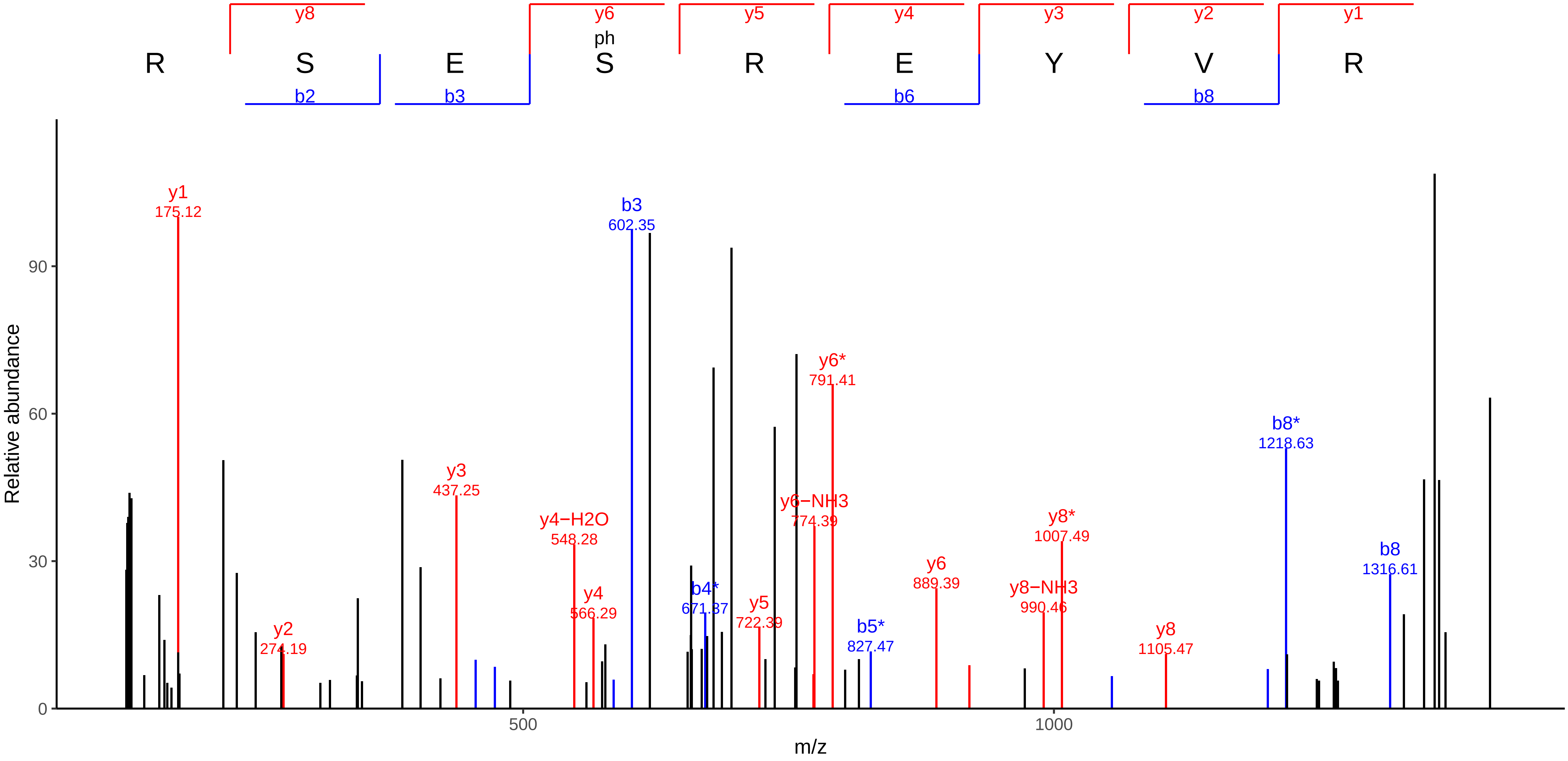

Supplement: Supplemental Table S11 — The key spectums of significantly changing proteins in Fig. 7. [file mmc11.pdf]
